# Achieving accelerated electrochemical kinetics in lithium–sulfur batteries by regulating the asymmetry factor *g* of chiral catalysts

**DOI:** 10.1093/nsr/nwag448

**Published:** 2026-07-22

**Authors:** Shixin Wang, Jinghan Wang, Jibing Tu, Yu Song, Shengzhe Ying, Yangyang Wu, Lize Wu, Hongpeng Li, Xiaojing Lin, Zhengqiang Hu, Hongsen Li

**Affiliations:** College of Physics, Qingdao University, Qingdao 266071, China; College of Physics, Qingdao University, Qingdao 266071, China; College of Physics, Qingdao University, Qingdao 266071, China; College of Physics, Qingdao University, Qingdao 266071, China; College of Physics, Qingdao University, Qingdao 266071, China; College of Physics, Qingdao University, Qingdao 266071, China; College of Physics, Qingdao University, Qingdao 266071, China; College of Mechanical Engineering, Yangzhou University, Yangzhou 225127, China; College of Physics, Qingdao University, Qingdao 266071, China; College of Physics, Qingdao University, Qingdao 266071, China; College of Physics, Qingdao University, Qingdao 266071, China

**Keywords:** Li–S batteries, chiral-induced spin selectivity effect, chiral asymmetric factor, electron spin manipulation, magnetic field

## Abstract

Manipulating electron spin is an effective approach for enhancing the activity of catalysts. However, progress remains limited due to the lack of stable and universal strategies. Here, we report an innovative strategy to regulate the spin state of Co_3_O_4_ that relies on the chiral-induced spin selectivity effect, whereby modulation of the chiral asymmetric factor *g* through an external magnetic field enables elucidation of its intrinsic correlation with catalytic performance. The experimental results demonstrate that chiral Co_3_O_4_ nanoparticles used as separator modifiers in lithium–sulfur (Li–S) batteries exhibit superior performance compared to achiral Co_3_O_4_. Moreover, we demonstrate a linear positive correlation between the *g* factor of chiral nanoparticles and the catalytic performance. Theoretical calculations reveal that the improved electrochemical performance primarily arises from an upshift of the Co *d*-band center, which effectively regulates interfacial charge-transfer kinetics. These findings provide guidance for the rational design of Li–S catalysts through electron spin engineering.

## Teaser

This study uncovers the role of the chiral-induced spin selectivity effect under magnetic field regulation in sulfur electrocatalysis for Li–S batteries, and establishes a correlation between the chiral asymmetry factor *g* and sulfur catalytic performance.

## INTRODUCTION

Owing to their high theoretical specific capacity (1675 mAh g^−1^) and high energy density (2600 Wh kg^−1^), lithium–sulfur (Li–S) batteries have emerged as highly attractive candidates for next-generation energy storage technologies [1–4]. However, the commercialization of Li–S batteries is mainly plagued by the severe shuttling of lithium polysulfides (LiPSs) and sluggish reaction kinetics [5,6]. Over the past decades, various electrocatalysts have been investigated to enhance the reaction kinetics, guided by the band theory [7–11]. Approaches including heteroatom doping and heterostructure engineering have been widely explored to tailor the electronic configuration, thereby regulating the adsorption behavior and catalytic conversion kinetics of active intermediates [12–16]. However, the applicability of current strategies across different metal elements and chemical compositions remains limited owing to the significant differences in catalytic effects among various elements. Therefore, establishing a stable and universally applicable strategy for manipulating the electronic structure remains an urgent need.

Recent studies discovered that chiral molecules have a preferred orientation of the electron spin due to the chiral-induced spin selectivity (CISS) effect (Fig. 1a), which has notable implications for electrocatalytic reactions [17–19]. In addition, the chiral asymmetric factor *g* characterizes the differential response of a material to left- and right-handed circularly polarized light. An increased *g* value generally signifies a stronger CISS effect, reflecting enhanced spin-selective electron transport (Fig. 1b) [20,21]. However, effective strategies for tuning the chiral asymmetry factor *g* remain unexplored in Li–S batteries. Therefore, establishing rational approaches for tuning the *g* factor is crucial for understanding its relationship with the catalytic performance of sulfur reduction.

**Figure 1.**
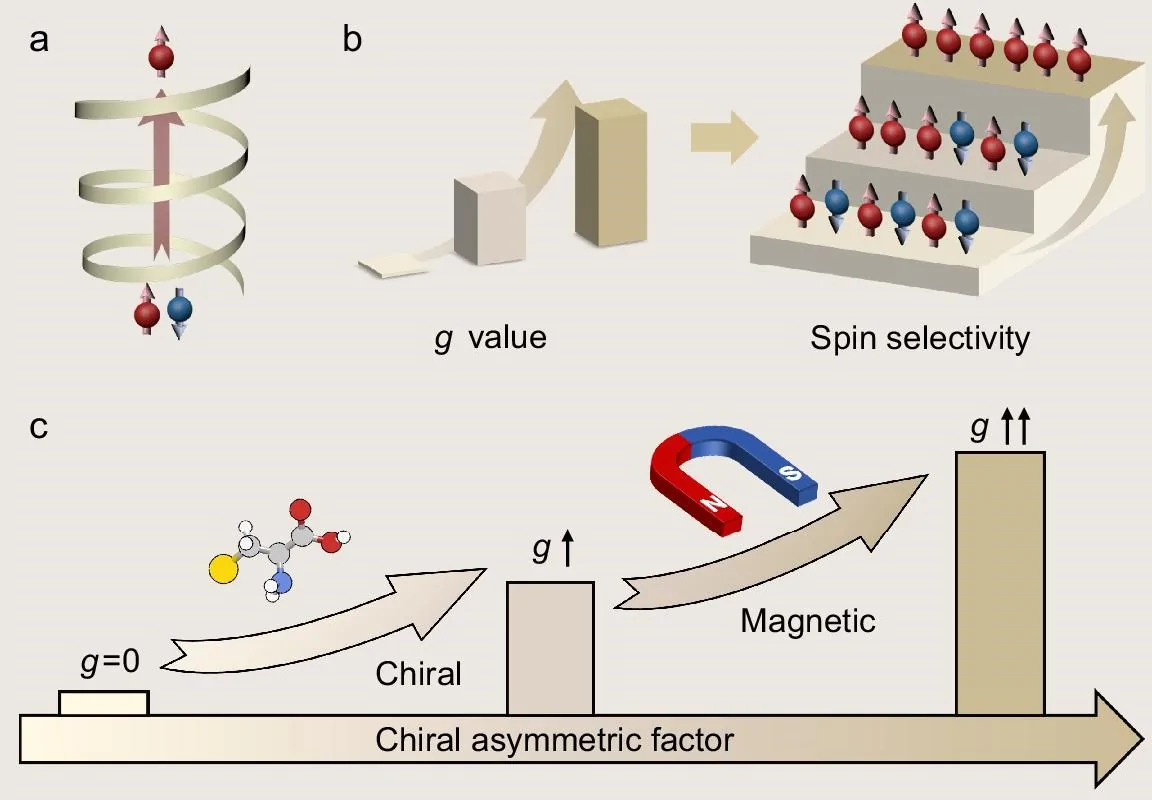
(a) CISS effect of electron transmission through chiral molecules. (b) The relationship between the *g* factor and electron spin selectivity. (c) Comparison of *g* factors under different conditions.

In this study, we report the realization of high-performance Li–S batteries using chiral-molecule-modified Co_3_O_4_ nanoparticles (NPs) as separator modification catalysts. We demonstrate that the CISS effect can significantly accelerate redox reactions in Li–S batteries. Furthermore, we investigated the relationship between the *g* factor and catalytic performance through an external magnetic field (Fig. 1c). The experimental results indicate that as the *g* factor increases, the overpotential of sulfur reduction continuously decreases, while the capacity of Li–S batteries shows consistent improvement. Density functional theory (DFT) calculations further revealed that a higher *g* factor corresponds to a higher spin state of Co. Along with *g* increase, the overlap of the electron density of states between the Co 3*d* orbitals and the S 3*p* orbitals also increases. This enhancement induces a stronger *d*–*p* hybridization, making electron transfer kinetically faster at the electrocatalytic sulfur reduction reaction.

## RESULTS AND DISCUSSION

Chiral Co_3_O_4_ NPs were prepared by employing D-, L- and DL-cysteine (Cys) enantiomers as surface ligands, denoted as D-Co_3_O_4_, L-Co_3_O_4_ and DL-Co_3_O_4_, respectively [22]. The sulfhydryl groups of Cys molecules coordinate with Co cations on the surface of the NPs, imparting chirality to the resulting Co_3_O_4_ NPs (detailed synthesis procedures are provided in the Experimental section in the supplementary data) [23]. The Co 2*p* X-ray photoelectron spectroscopy (XPS) spectra (Fig. S1) reveal that L-Co_3_O_4_ NPs show the characteristic peaks of Co oxidation state located at 781.3, 797.3 eV (Co^2+^) and 780.3, 795.3 eV (Co^3+^), respectively, and two corresponding shake-up satellite peaks [24–26]. This observation indicates the presence of Co ions exhibiting mixed valence states.

Moreover, X-ray absorption near-edge structure (XANES) and Fourier transform extended X-ray absorption fine structure (FT-EXAFS) analyses were conducted to investigate the electronic structure and coordination environment of chiral Co_3_O_4_ NPs. The Co *K*-edge XANES spectra (Fig. 2a) of L-Co_3_O_4_ NPs are located close to the Co_3_O_4_ reference sample at ∼7715 eV, further suggesting the valence state of Co [27]. The FT-EXAFS spectra (Fig. 2b) of L-Co_3_O_4_ NPs show two main peaks corresponding to the Co–O and Co–Co coordination shells, respectively. However, the primary peak of L-Co_3_O_4_ NPs, located at ∼1.5 Å [28], exhibits a slight shift toward higher *R* values in comparison to the reference, which could be attributed to the formation of Co–S bonds between the surface Co atoms and Cys molecules. The similar bond lengths of Co–S and Co–O result in overlapping signals in R space, complicating the complete distinction between the two [29]. To further visualize the coordination environment of Co atoms, a wavelet transform (WT) analysis of the FT-EXAFS data was conducted (Fig. 2c). The results confirm the coexistence of Co–O, Co–S and Co–Co coordination structures in L-Co_3_O_4_.

**Figure 2.**
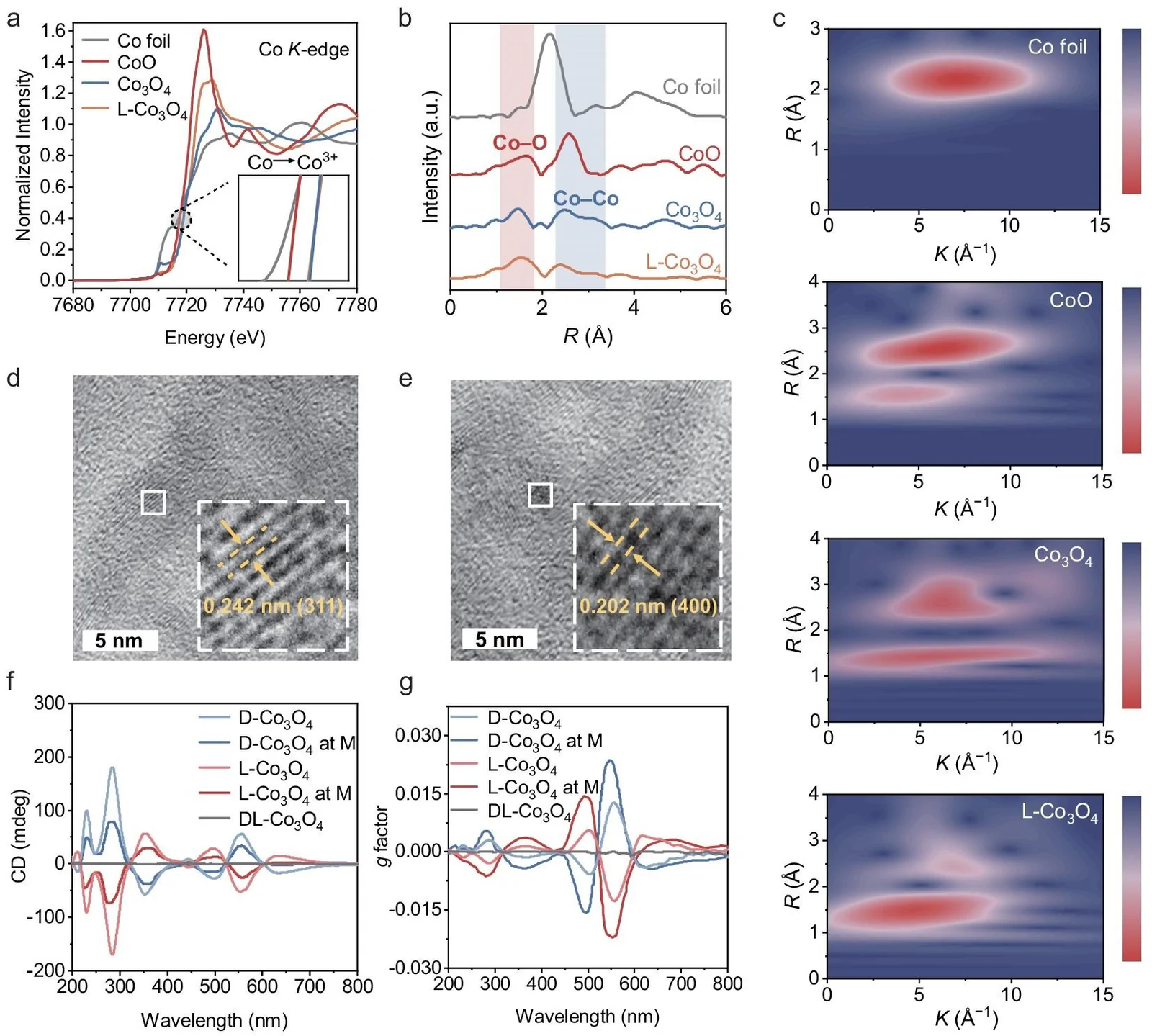
(a) Co *K*-edge XANES spectra of L-Co_3_O_4_ and other reference materials (inset shows the magnified XANES spectra). (b) FT-EXAFS spectra of L-Co_3_O_4_ and other reference materials. (c) WT plots of the EXAFS signals for L-Co_3_O_4_ and other reference materials. (d and e) HRTEM images of L-Co_3_O_4_. (f) CD spectra, and (g) *g* factors of Co_3_O_4_ NPs stabilized by D-Cys, L-Cys and DL-Cys (M denotes the magnetic field).

Moreover, the high-resolution transmission electron microscopy (HRTEM) images of the L-Co_3_O_4_ NPs reveal clear lattice fringes, and the corresponding lattice spacings of 0.202 and 0.242 nm can be indexed to the (400) and (311) planes, respectively (Fig. 2d and e) [30]. Low-magnification TEM images show that DL-Co_3_O_4_ and L-Co_3_O_4_ exhibit comparable particle sizes and similar NP geometries, suggesting that morphology differences are unlikely to be the primary origin of the observed electrochemical performance variation (Fig. S2). The NPs exhibit an average size of ∼5 nm. Figures 2f and S3 illustrate the circular dichroism (CD) and ultraviolet-visible absorption spectra of the NPs. The absorption bands centered at 230, 280 and 350 nm are commonly assigned to Co(II)→Co(III) intraparticle transitions, whereas those located at 450, 550 and 600 nm are attributed to transitions from surface states (including ligand-related states) to Co(III) [30]. The enhanced CD response in the 230–350 nm region suggests that the chiral environment modifies the symmetry of the Co-related electronic transitions, leading to stronger chiroptical activity of the Co_3_O_4_ NP system. Figures 2g and S4 illustrate the variation of the *g* factor of the NPs. The results indicate that the *g* factor of achiral Co_3_O_4_ (DL-Co_3_O_4_) is the smallest, approaching zero, while the chiral Co_3_O_4_ samples (D-Co_3_O_4_ and L-Co_3_O_4_) demonstrate the largest *g* factors under an external magnetic field. It should be noted that the dissymmetry factor is used here as an experimental descriptor of chiral asymmetry rather than a direct measure of the presence or concentration of chiral molecules. To assess the spin selectivity of the synthesized chiral Co_3_O_4_ NPs, magnetic conductive atomic force microscopy measurements were performed. The average current–voltage curves of L-Co_3_O_4_ NPs were recorded under different magnetization orientations (tip up and tip down), as shown in Fig. S5. Significantly higher currents were observed when the tip was magnetized upward compared with the downward magnetization. This result suggests a preferential transfer of electrons whose spin orientation is parallel to the tip magnetization, rather than those with an antiparallel spin configuration. It should be noted that the present study focuses on chiral environments introduced during material synthesis, and whether post-synthesis molecular modification or ligand exchange can reproduce the same effects remains an open question for future investigation.

To investigate the role of the CISS effect in Li–S batteries, we systematically examined the electrochemical performance of chiral and achiral Co_3_O_4_ NPs utilized as the polypropylene (PP) separator modifiers. The separator was selected as the catalyst-supporting platform because it enables direct regulation of dissolved polysulfide transport and conversion while minimizing perturbation to the sulfur electrode composition and architecture, thereby allowing the spin-dependent catalytic effect to be evaluated more independently. From the thermogravimetry curve in Fig. S6, the sulfur content in the S/C cathode is 74.5%. The modified separator is shown in Fig. S7. Although the introduction of Co_3_O_4_ NPs increases the inactive mass of the battery, the relatively low loading and the resulting improvement in sulfur utilization and reaction kinetics partially offset the potential reduction in practical energy density. Linear sweep voltammetry (LSV) was performed to evaluate the electrochemical stability of the chiral Co_3_O_4_ system in the Li–S battery electrolyte (Fig. S8). Chiral Co_3_O_4_ NPs were loaded as the working electrode material, and the LSV results show that no obvious additional electrochemical processes occurred within the operating voltage window, indicating sufficient electrochemical stability of the chiral Co_3_O_4_ system under the electrolyte and electrochemical conditions employed. Figure 3a presents the galvanostatic charge–discharge (GCD) curves at 0.1 C. The discharge profiles exhibit two distinct plateaus (Q_1_ and Q_2_), corresponding to the reduction of sulfur to soluble LiPSs and the subsequent transformation to insoluble products, respectively. In general, the experimentally determined Q_2_/Q_1_ ratio serves as an indicator of the extent of the reduction process and the suppression of LiPSs diffusion, which is closely related to the catalytic activity. The results indicate that the Q_2_/Q_1_ ratios for the batteries with D-Co_3_O_4_-PP and L-Co_3_O_4_-PP are 2.60 and 2.56, respectively. Both of these values are significantly higher than the ratio observed for the battery with DL-Co_3_O_4_-PP, which is 2.33.

**Figure 3.**
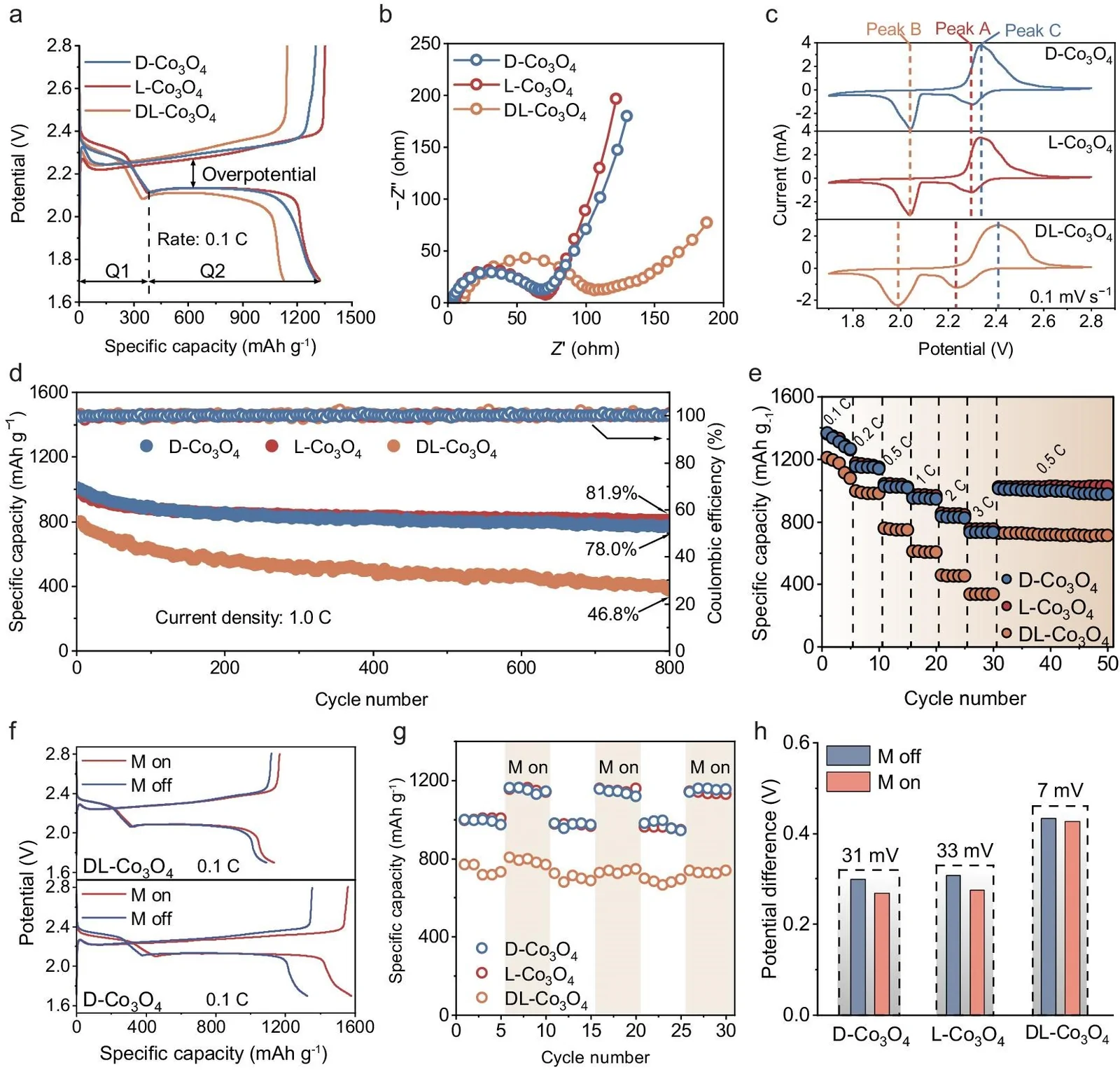
(a) Initial charge–discharge curves at a current rate of 0.1 C. (b) Nyquist plots. (c) CV curves at a scan rate of 0.1 mV s^−1^. (d) Cycling performance at 1 C for 800 cycles. (e) Rate performance at various current densities. (f) Charge–discharge curves of L-Co_3_O_4_ and DL-Co_3_O_4_ in the presence of the magnetic field. (g) Specific capacity changes in the presence of the magnetic field. (h) Comparison of the potential difference between the oxidation peak and the reduction peak in the presence of the magnetic field.

Figure 3b shows the electrochemical impedance spectra (EIS). The Nyquist plots display semicircles in the high-frequency region and nearly linear behavior in the low-frequency region. The semicircle in the high-frequency region corresponds to the charge-transfer resistance, where a smaller semicircle indicates lower resistance; the slope of the low-frequency line is related to ion diffusion resistance, with a larger slope implying faster ion diffusion. As observed, the batteries with D-Co_3_O_4_-PP and L-Co_3_O_4_-PP exhibit significantly smaller semicircles and steeper low-frequency slopes. The curves of the batteries with D-Co_3_O_4_-PP and L-Co_3_O_4_-PP almost overlap, indicating comparable charge-transfer and diffusion resistances for both, which are much lower than those of DL-Co_3_O_4_ demonstrating superior electrochemical reaction kinetics. Figure 3c shows the cyclic voltammetry (CV) curves of different Li–S batteries recorded at a scan rate of 0.1 mV s^−1^ within a potential window of 1.7–2.8 V. All samples exhibit two typical reduction peaks and one oxidation peak, consistently with GCD data. Evidently, a positive shift in the reduction peaks and a negative shift in the oxidation peaks of the batteries with D-Co_3_O_4_-PP and L-Co_3_O_4_-PP indicate the improved redox kinetics compared with DL-Co_3_O_4_-PP.

Tafel plots are employed to assess the catalytic performance of electrochemical reactions and to analyze their reaction kinetics. Figure S9 presents the Tafel plots derived from both the oxidation and reduction processes. The batteries with D-Co_3_O_4_-PP and L-Co_3_O_4_-PP exhibit nearly identical and relatively small Tafel slopes during oxidation and reduction processes, illustrating the high catalytic conversion activity for sulfur species. The specific capacity of the batteries with chiral Co_3_O_4_-PP remains ∼800 mAh g^−1^ under a current density of 1 C with a capacity retention of ∼81% after 800 cycles (Fig. 3d), demonstrating a superior electrochemical performance compared to the battery with DL-Co_3_O_4_ -PP. Figure 3e shows the rate capabilities of the batteries with chiral and achiral Co_3_O_4_-PP, with the rates varying from 0.1 to 3 C. Obviously, the batteries with chiral Co_3_O_4_-PP exhibit superior rate performance compared to the achiral Co_3_O_4_, demonstrating that the chiral Co_3_O_4_ with the CISS effect exhibits significantly enhanced electrochemical performance.

To further investigate the influence of the increased *g* factor on the catalytic performance of Li–S batteries, we compared the electrochemical behaviors of chiral and achiral Co_3_O_4_ NPs used as separator modification catalysts in the presence and absence of an external magnetic field. The Co_3_O_4_ NPs and L-Co_3_O_4_ NPs both exhibit paramagnetic behavior (Fig. S10), with their magnetic moments displaying only very small variation under an external magnetic field. Consequently, their electrochemical performances are minimally affected by the applied magnetic field [31]. The device used for generating the magnetic field is illustrated in Fig. S11. Figures 3f and S12 present the GCD curves of the batteries with chiral and achiral Co_3_O_4_-PP at a rate of 0.1 C in the presence and absence of the external magnetic field. To further evaluate the intrinsic contribution of the magnetic field, first-cycle charge–discharge measurements were conducted using untreated PP separators under identical conditions. Only a slight reduction in polarization was observed after applying the magnetic field, indicating that conventional magnetic-field-induced effects contribute only marginally to the overall electrochemical improvement. The batteries with chiral Co_3_O_4_-PP exhibit a significantly higher initial specific capacity and a lower overpotential under the external magnetic field, while the DL-Co_3_O_4_ shows only a slight improvement in capacity and a minor change in overpotential. In the EIS as shown in Fig. S13, the batteries with chiral Co_3_O_4_-PP display a much smaller high-frequency semicircle and a steeper low-frequency slope under the external magnetic field, indicating significantly reduced charge-transfer and diffusion resistances. The more pronounced impedance-profile variation observed for DL-Co_3_O_4_ under the magnetic field is mainly attributed to the magnetohydrodynamic effect on ion transport, whereas in the chiral Co_3_O_4_ systems the magnetic field predominantly enhances spin-selective interfacial charge-transfer kinetics associated with the CISS effect. Figure 3g shows the variation in specific capacity at a current density of 0.5 C when the batteries were alternately exposed to and removed from the magnetic field. The results reveal that the specific capacity of the batteries with D-Co_3_O_4_-PP and L-Co_3_O_4_-PP changes significantly with the magnetic field on–off cycles, while that of DL-Co_3_O_4_ remains almost unchanged, suggesting that the electrochemical behavior of the chiral system is highly sensitive to the external magnetic field. Figure S14 compares the CV curves of batteries with different catalysts in the presence and absence of the magnetic field and at a scan rate of 0.1 mV s^−1^. It is evident that the batteries with chiral Co_3_O_4_-PP exhibit sharper redox peaks, stronger peak currents and overall reduced overpotential in the presence of the magnetic field. Figure 3h quantitatively analyzes the potential difference between oxidation peak and reduction peak. Under the influence of the magnetic field, the potential difference of the batteries with D-Co_3_O_4_-PP and L-Co_3_O_4_-PP decreases by 31 and 33 mV, respectively, while the potential difference of DL-Co_3_O_4_ decreases by only 7 mV. The battery with DL-Co_3_O_4_-PP also exhibited a slight improvement, likely due to the magnetohydrodynamic convection effect that facilitates ion transport [32,33]. As illustrated in Fig. 3, a higher *g* factor reflects improved electrochemical performance. Noteworthily, although D-Co_3_O_4_ and L-Co_3_O_4_ filter electrons with opposite spin orientations, their catalytic performances do not exhibit significant differences. This can be attributed to the fact that the spin orientation of electrons is an artificially defined reference direction rather than a fixed orientation within the molecular framework [18]. Consequently, the two enantiomers exhibit essentially identical catalytic activity, which is consistent with the experimental results. Based on these findings, L-Co_3_O_4_ has been selected as a representative system for the subsequent discussion.

Potentiostatic discharge experiments were conducted to analyze the reaction kinetics of Li_2_S deposition (Fig. 4a–c) [34,35]. For quantitative comparison, the Li_2_S precipitation capacities were calculated from the integrated potentiostatic current and normalized, based on the sulfur content in the Li_2_S_8_ catholyte. Under an applied magnetic field, the battery with L-Co_3_O_4_-PP delivers the highest Li_2_S nucleation capacity (238.6 mAh g^−1^) and the earliest current response (1330 s), indicating enhanced electrocatalytic activity toward sulfur oxidation (Table S1). In addition, the electrocatalytic performance for LiPSs conversion was further examined by CV measurements of symmetric cells. With the magnetic field applied, the L-Co_3_O_4_ symmetric cell exhibits the highest peak current, the smallest polarization and the largest peak area, demonstrating superior catalytic capability for LiPSs conversion (Fig. 4d). Moreover, *in situ* and *ex situ* Raman spectroscopy were conducted to probe the LiPSs shuttle behavior. After several cycles, Raman peaks assigned to S_8_^2−^ at 150, 218 and 476 cm^−1^ appear at the first discharge plateau and persist until the end in the DL-Co_3_O_4_ battery. Meanwhile, characteristic signals corresponding to S_6_^2−^ and S_5_^2−^ + S_4_^2−^ emerge at 399 and 474 cm^−1^, respectively, indicating the occurrence of a pronounced shuttle effect (Fig. 4e) [36]. In contrast, in the L-Co_3_O_4_ battery, a much lower intensity of LiPSs was detected throughout the entire discharging process, demonstrating its outstanding LiPSs-blocking capability (Fig. 4f). Because the initial Raman intensity of S_8_ differs between the two cells, the Raman comparison should be interpreted mainly in terms of the spectral evolution trend during discharge rather than the absolute signal intensity at a single state. Figure S15 shows the Raman spectra collected from disassembled cells after discharge to the corresponding voltages in the presence and absence of an external magnetic field. The results indicate that the Raman signal intensity of L-Co_3_O_4_ in the presence of an external magnetic field is significantly weaker than that observed in its absence. This finding further corroborates that the magnetic field enhances the adsorption capacity of L-Co_3_O_4_ for LiPSs. In summary, as the asymmetric factor *g* increases, the shuttle effect is gradually suppressed, and the catalytic performance of the catalyst is continuously improved (Fig. 4g).

**Figure 4.**
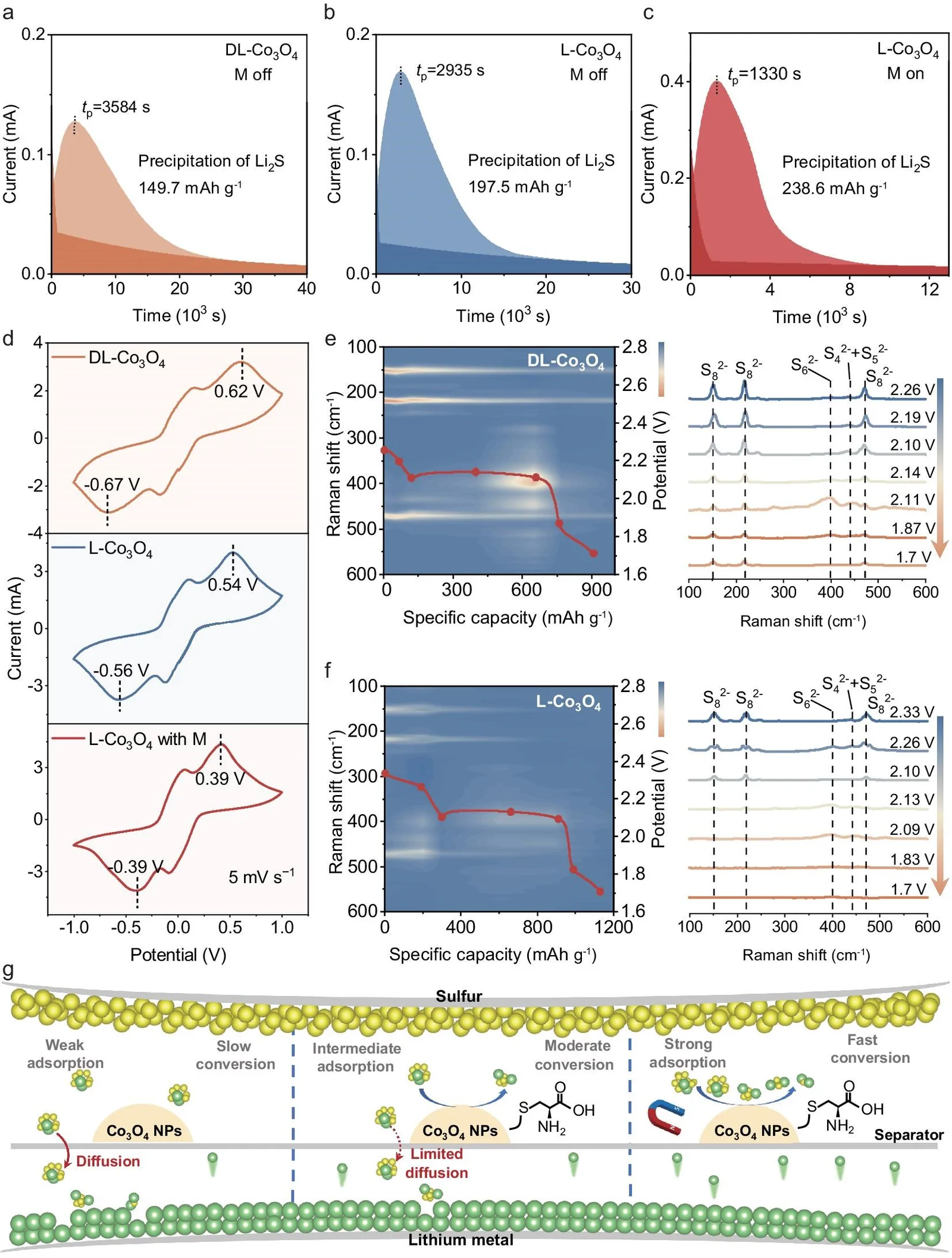
Li_2_S precipitation curves of (a) DL-Co_3_O_4_, (b) L-Co_3_O_4_ and (c) L-Co_3_O_4_ with magnetic field. (d) CV curves of symmetric cells. *In situ* Raman spectra of (e) DL-Co_3_O_4_ and (f) L-Co_3_O_4_. (g) Enhancing the *g* factor can effectively suppress the shuttle effect of LiPSs and improve the catalytic performance.

It should be noted that the role of the catalyst is not to permanently immobilize polysulfides, but to promote dynamic adsorption and catalytic conversion during sulfur redox reactions. Excessive accumulation of sulfur species or solid Li_2_S_2_/Li_2_S near the separator would be expected to induce ion-transport limitations, increased polarization and rapid capacity decay. However, the reduced charge-transfer/diffusion resistance and stable long-term cycling behavior observed for the chiral Co_3_O_4_-modified cells suggest that such blocking effects remain limited under the present conditions. Compared with direct cathode modification, separator-side catalyst regulation provides an additional spatial interface to intercept migrating polysulfides before they reach the Li metal anode, while maintaining the original cathode composition and sulfur-loading architecture. Therefore, this strategy should be regarded as complementary to, rather than a replacement for, cathode-side catalyst design.

To further explore the catalytic mechanism of the chiral asymmetric factor *g* in electrochemical reactions, DFT calculations were conducted on the chiral structures. It is noteworthy that current descriptions of the CISS effect based on first-principles methods significantly underestimate conductance and spin splitting by several orders of magnitude, primarily due to a limited understanding of the CISS phenomenon itself [37–39]. This discrepancy mainly stems from an incomplete theoretical comprehension of the CISS effect, with deviations often attributed to factors such as phase mismatch and local charge leakage. In spintronics research, such effects are typically described using nonequilibrium spin accumulation models or the Büttiker probe approach [40,41]. However, integrating these complex spin transport processes into calculations poses significant technical challenges and exceeds the scope of this work. Therefore, this study primarily focuses on elucidating the catalytic mechanism of electrochemical reactions within chiral systems.

It should be noted that the experimentally measured *g* factor was not explicitly calculated or directly incorporated into the present DFT models. Likewise, neither the external magnetic field nor the CISS-induced spin-filtering process was explicitly simulated. Therefore, the DFT calculations should be regarded as simplified electronic-structure models representing different experimentally observed spin-polarization states. The calculated spin density distributions, projected density of states (PDOS), adsorption energies and Gibbs free-energy profiles provide a possible electronic-structure interpretation that is consistent with the experimentally observed correlation between increased *g* factor and enhanced sulfur redox kinetics.

Through DFT calculations, the influence of the *g* factor on the electronic structure of Co_3_O_4_ was elucidated. As illustrated by the spin density distributions of Co_3_O_4_ (Fig. 5a), an increase in the *g* factor, in comparison to DL-Co_3_O_4_, results in elevated spin density and a greater accumulation of spin electrons around the Co atoms. The magnetic moment of Co atoms increases with the degree of spin polarization, leading to electron redistribution and a transition in spin states [42,43]. Furthermore, the spin-up orbital is characterized by higher energy than the spin-down orbital, which causes an upward shift in the *d*-band center, thereby enhancing the adsorption strength between the catalytic sites and LiPSs [44]. Figure 5b presents the PDOS of Co_3_O_4_ with varying *g* factors following the adsorption of Li_2_S_4_. As the *g* factor increases, the overlap between the Co 3*d* and S 3*p* orbitals intensifies, resulting in enhanced *d*–*p* hybridization and more efficient charge-transfer kinetics between the two species. The calculated Gibbs free energy profiles are illustrated in Figs 5c and S16. For clarity, the chiral molecular species are omitted in the displayed structures, and only the adsorption configurations of Li_2_S_x_ on Co_3_O_4_ are shown. It should be noted that stronger adsorption is not universally beneficial for all sulfur intermediates. The relatively enhanced interaction with Li_2_S_2_/Li_2_S may facilitate the nucleation and stabilization of solid discharge products during the final sulfur reduction stage. With an increasing *g* factor, the energy barriers for each elementary reaction step progressively decrease. This observation indicates that a higher *g* factor facilitates thermodynamically favorable reduction processes, promoting smoother transformations among intermediates and accelerating overall reaction kinetics. Figure S16 demonstrates that a higher *g* factor correlates with stronger adsorption energy toward sulfur species, effectively enhancing the catalytic conversion of LiPSs. As the *g* factor increases, the spin state of the catalytic sites progressively rises, which not only accelerates electron transfer between the catalytic sites and LiPSs but also reduces the energy barrier of the sulfur redox reaction, thereby significantly improving the catalytic performance of Li–S batteries.

**Figure 5.**
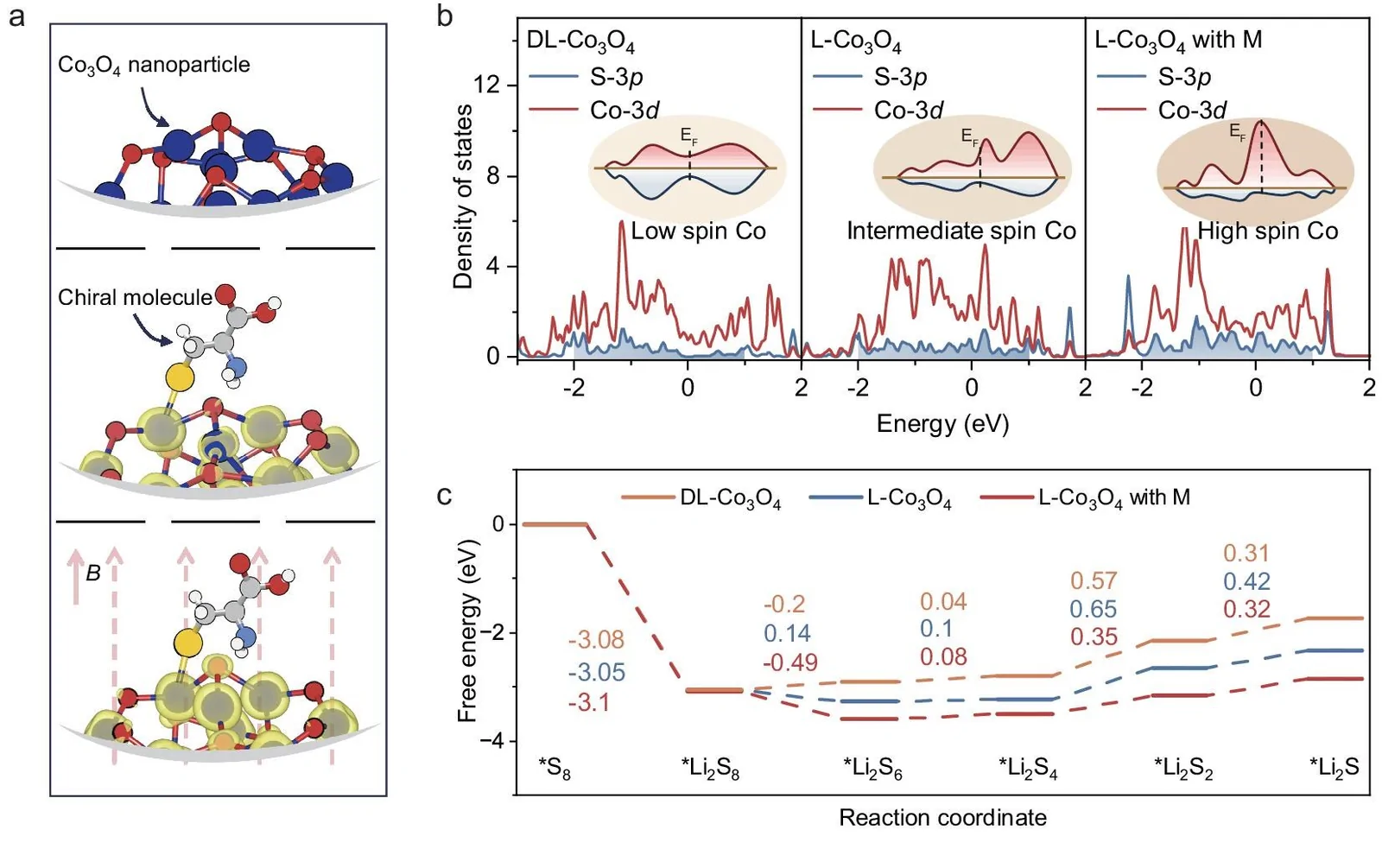
(a) Spin density of DL-Co_3_O_4_, L-Co_3_O_4_ and L-Co_3_O_4_ in the presence of magnetic field. (b) PDOS of DL-Co_3_O_4_, L-Co_3_O_4_ and L-Co_3_O_4_ under magnetic field interacting with Li_2_S_4_. (c) Gibbs free energy.

## CONCLUSION

In summary, we have demonstrated that the CISS effect can significantly enhance the kinetic and stability performance of Li–S batteries. The chiral Co_3_O_4_ serves as an effective spin polarizer, and the chiral asymmetry factor *g* can be tuned by an external magnetic field to regulate the CISS effect. As *g* increases, enhanced spin polarization and an upshifted Co *d*-band center strengthen LiPSs adsorption and accelerate redox kinetics, thereby improving the catalytic performance of Li–S batteries. DFT calculations reveal that an increased *g* factor results in a higher spin state of Co catalytic sites and a larger number of unpaired electrons, thereby strengthening the interaction with LiPSs. Consequently, Li–S batteries demonstrate continuous improvements in specific capacity, rate capability and cycling stability. This work provides a novel and feasible approach to the design of a transition metal-based catalyst by modulating the chiral asymmetric factor *g*.

## MATERIALS AND METHODS

### Synthesis of cobalt oxide NPs

Co_3_O_4_ NPs were synthesized using cobalt(II) chloride as the metal precursor and Cys as the surface ligand in an aqueous medium. Briefly, 15 mL of deionized water, 2.5 mL of 100 mM NaBH_4_, 2 mL of 100 mM L/D/DL-Cys, 2 mL of 100 mM sodium citrate and 1 mL of 200 mM cobalt(II) chloride were sequentially added into a round-bottom flask and magnetically stirred for 2 h at room temperature (23–25°C). During the reaction, the solution gradually turned transparent dark brown, indicating the formation of Co_3_O_4_ NPs. The resulting NPs were precipitated by introducing isopropanol at seven times the volume of the reaction solution, followed by centrifugation at ∼10 000 G for 20 min to achieve purification. During synthesis, Cys molecules are proposed to interact with cobalt precursor species and regulate the local surface configuration formed during NP growth, resulting in distinct chiral characteristics of the obtained Co_3_O_4_ NPs.

### Preparation of modified separators and sulfur cathodes

L/D/DL-Co_3_O_4_-modified separators were prepared via a straightforward surface-coating method. Briefly, 70 wt.% of the active material, 20 wt.% carbon black (Super P) and 10 wt.% polyvinylidene fluoride (PVDF) were dispersed in 1-methyl-2-pyrrolidinone (NMP) and vigorously stirred to form a homogeneous slurry. The resulting slurry was uniformly cast onto a pristine Celgard 2500 PP separator. The coated membranes were dried under vacuum at 50°C overnight and subsequently punched into circular discs with a diameter of 19 mm. The areal mass loading of the interlayer was ∼0.3 mg cm^−2^. The sulfur/carbon nanotube (S/CNT) composite was prepared by a melt-diffusion process. CNTs and sulfur powder were mixed at a weight ratio of 1:3 and then heated at 155°C for 12 h. The sulfur loading in the cathodes was ∼1.0 mg cm^−2^.

### Li–S battery assembly and electrochemical performance measurements

The working electrodes were prepared by mixing 80 wt.% S/CNT active material, 10 wt.% Super P and 10 wt.% PVDF in NMP to form a uniform slurry, which was then cast onto carbon-coated aluminum foil. The electrodes were dried in a vacuum oven at 60°C overnight. Electrochemical performance was evaluated using CR2032 coin-type half cells. The Li–S cells were assembled in an argon-filled glove box (H_2_O < 0.1 ppm, O_2_ < 0.1 ppm), employing sulfur cathodes, lithium metal anodes and the modified separators. The electrolyte consisted of 1,3-dioxolane and dimethoxymethane (1:1 by volume), containing 1.0 M lithium bis(trifluoromethanesulfonyl)imide and 1 wt.% LiNO_3_ as an additive. The electrolyte-to-sulfur ratio was controlled at 15 μL mg^−1^ for cathodes with an areal sulfur loading of ∼1.0 mg cm^−2^. GCD tests were conducted on a NEWARE battery testing system (CT-4008T-5V20 mA-164, Shenzhen, China) at room temperature within a voltage window of 1.7–2.8 V. CV and EIS measurements were carried out using an electrochemical workstation (IVIUM Technologies, Vertex.One.EIS) over a frequency range of 0.01 Hz to 100 kHz. EIS measurements were conducted after cell assembly and a resting period under open-circuit conditions prior to the first charge–discharge cycle. Notably, the 304 stainless steel components of the coin cells possess an austenitic structure and are non-ferromagnetic; therefore, they cannot be magnetized and do not interfere with the applied magnetic field.

## Supplementary Material

nwag448_Supplemental_File
