# Supplementary material for: Achieving accelerated electrochemical kinetics in lithium–sulfur batteries by regulating the asymmetry factor g of chiral catalysts
Source: Natl Sci Rev. 2026 Jul 22;13(18):nwag448. doi: 10.1093/nsr/nwag448 (PMC13637589; doi:10.1093/nsr/nwag448)
Supplement: Supplementary file 1 [file nwag448_supplemental_file.pdf]

Supporting Information

Achieving accelerated electrochemical kinetics in lithium–sulfur batteries by regulating the asymmetry factor *g* of chiral catalysts

Shixin Wang[1,†], Jinghan Wang[1,†], Jibing Tu[1,†], Yu Song[1], Shengzhe Ying[1], Yangyang Wu[1], Lize Wu[1], Hongpeng Li[2], Xiaojing Lin[1], Zhengqiang Hu[1] and Hongsen Li[1,*]

[1]College of Physics, Qingdao University, Qingdao 266071, China;

[2]College of Mechanical Engineering, Yangzhou University, Yangzhou 225127, China.

***Corresponding author.** Email: hsli@qdu.edu.cn

†Equally contributed to this work.

Experimental Section

Materials and reagents

All chemical reagents are used as received without further purification. L/D/DL-Cysteine (Aladdin ≥ 98%), cobalt (II) chloride hexahydrate ($CoCl_2 \cdot 6H_2O$, Aladdin ≥ 98%), $NaBH_4$ (Aladdin, AR), sodium citrate ($C_6H_5Na_3O_7$, Aladdin ≥ 98%).

Materials characterizations

Transmission electron microscopy (JEOL, JEM-2100F) was employed to characterize the structure and morphology of the products. X-ray photoelectron spectroscopy (XPS, ESCALAB 250Xi) was conducted to investigate the valence states and surface chemical composition. Circular dichroism (CD) and UV-vis spectra were recorded on a JASCO J-1500 spectrometer over a scanning range of 190–800 nm under high-purity nitrogen. All measurements were performed at 25°C.

mc-AFM Measurements

The sample preparation procedure for mc-AFM was identical to that used for the CD measurements. I–V measurements were performed on an SPA-400 AFM system equipped with a NanoNavi IIe controller. CoCr tips (MESP-V2, Bruker) were premagnetized for 15 min in different magnetic field orientations, namely magnetic north (Tip Up) and south (Tip Down), using a strong permanent magnet. The premagnetized tips were then immediately applied to acquire I–V curves of the three samples in contact mode. Relatively flat regions with a typical roughness of ~200 nm were selected, and the voltage was swept from −2 to +2 V. The reported data represent averaged I–V curves.

Symmetric cell assembly and measurements

The symmetric cell electrodes were fabricated by dispersing 70 wt.% of the active sample, 20 wt.% Super P, and 10 wt.% polyvinylidene fluoride (PVDF) in N-methyl-2-pyrrolidinone (NMP) to obtain a uniform slurry, which was subsequently coated onto aluminum foil. Two identical electrodes were assembled as the working and counter electrodes. The electrolyte (40 μL) consisted of 0.5 mol L^{-1} Li_2S_6 and 1 M LiTFSI dissolved in a DOL/DME mixture (v/v = 1:1). For comparison, symmetric cells containing 1 M LiTFSI in DOL/DME (v/v = 1:1) without Li_2S_6 were also assembled in CR2032 coin-type cells and evaluated under identical conditions. Cyclic voltammetry (CV) measurements were conducted at a scan rate of 5 mV s^{-1} over a potential window ranging from −1.0 to 1.0 V.

Measurement of nucleation of Li_2S

Li_2S nucleation was examined in a CR2032 coin-type cell to evaluate the liquid–solid reaction kinetics. A D/L/DL-Co_3O_4 electrode without sulfur loading and lithium foil were used as the working and counter electrodes, respectively. The catholyte (20 μL) comprised 0.25 M Li_2S_8 and 1.0 M LiTFSI dissolved in a DOL/DME mixture (v/v = 1:1), whereas the anolyte (20 μL) contained 1.0 M LiTFSI in the same solvent without Li_2S_8. The cell was first galvanostatically discharged to 2.06 V at a current of 0.112 mA, followed by a potentiostatic step at 2.05 V until the current density declined below 10^{-2} A, enabling Li_2S nucleation and subsequent growth.

Computational methods

Calculations based on first-principles density functional theory (DFT), including molecular dynamics simulations were executed utilizing the Vienna Ab initio Simulation Package (VASP) in conjunction with the Projector Augmented Wave (PAW) methodology. The exchange-correlation functional was managed within the parameters of the Generalized Gradient Approximation (GGA), adopting the Perdew-Burke-Ernzerhof (PBE) functional. The long-range van der Waals interactions are accounted for through the DFT-D3 approach. We implemented a plane wave basis set with an energy cutoff set at 500 eV, and the geometric relaxation was carried through until the forces acting on each atom were less than 0.03 eV/Å. The sampling of the Brillouin zone was conducted using a 1 × 1 × 1 k-point grid. To assure rigorous consistency, calculations were performed until the energy convergence threshold was less than 10^{-5} eV. The free energy of the intermediates is calculated:

$$\Delta G = \Delta EDFT + \Delta ZPE - T\Delta S$$

Where ΔEDFT, ΔZPE and ΔS are the changes of the reaction energy obtained from DFT calculations, zero-point energy, and the changes of entropy from the initial state to the final state, respectively. T is temperature and the T of 298.15 K was used in all computations.

Table S1. Kinetic parameters derived from Li_2S precipitation experiments for DL-Co_3O_4, L-Co_3O_4, and L-Co_3O_4 under a magnetic field.

Sample	Nucleation time (s)	Peak current (mA)	Li_2S precipitation capacity (mAh g^{-1})
DL-Co_3O_4	3584	0.131	149.7
L-Co_3O_4	2935	0.172	197.5
L-Co_3O_4+M	1330	0.395	238.6

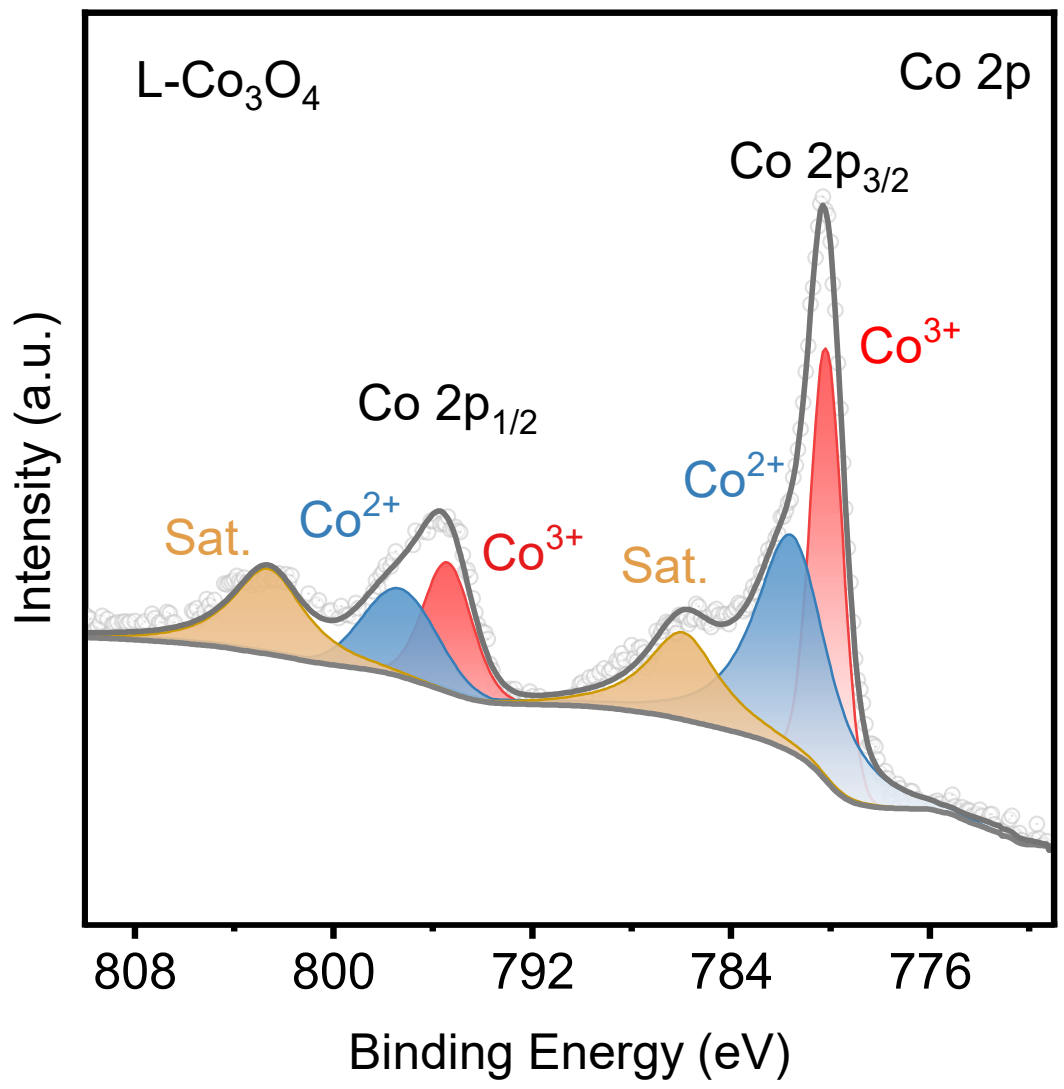

Figure S1. Co 2p region XPS spectra of L-Cys Co_3O_4 NPs.

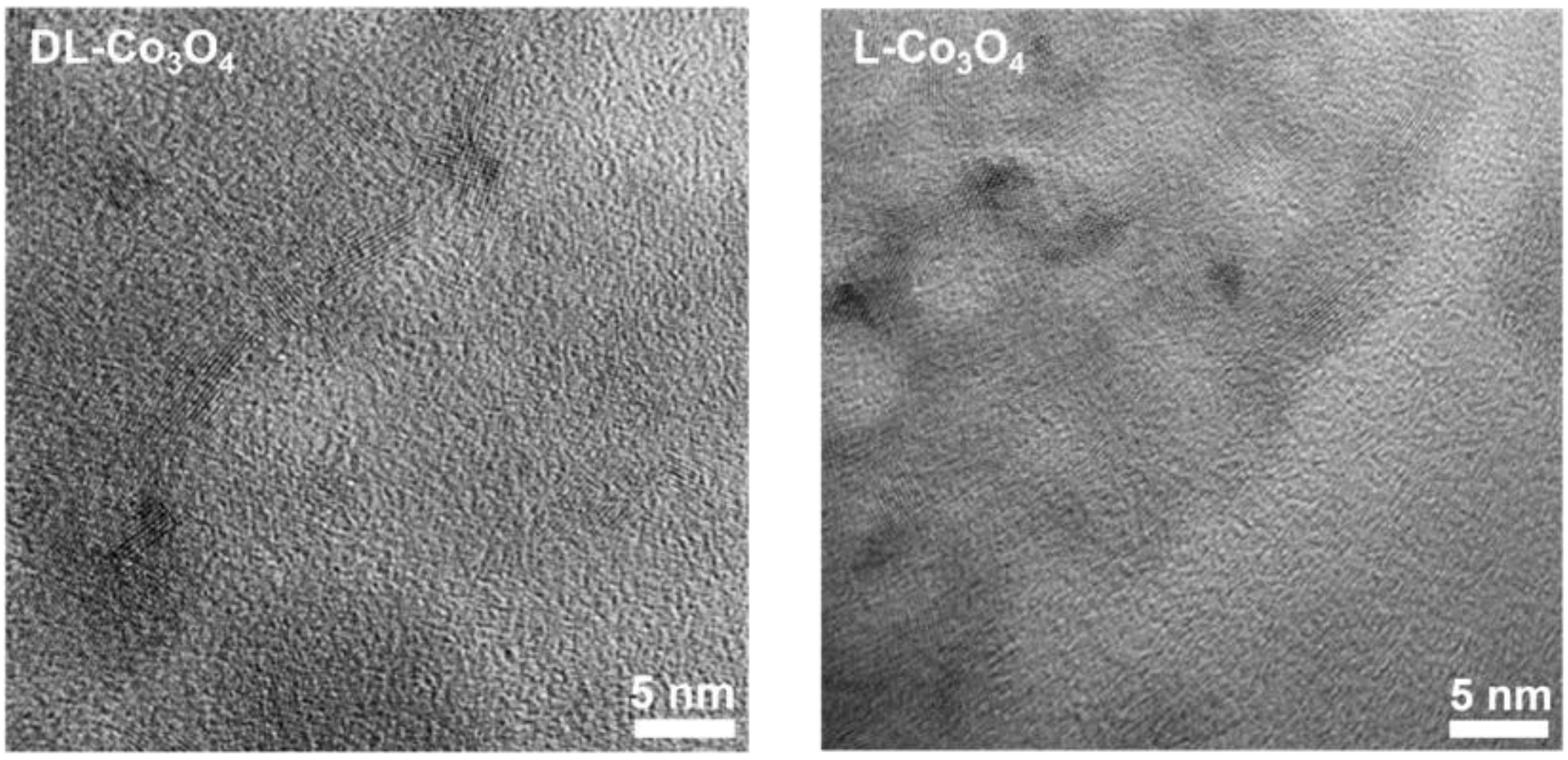

Figure S2. TEM images of DL-Co_3O_4 and L-Co_3O_4 NPs.

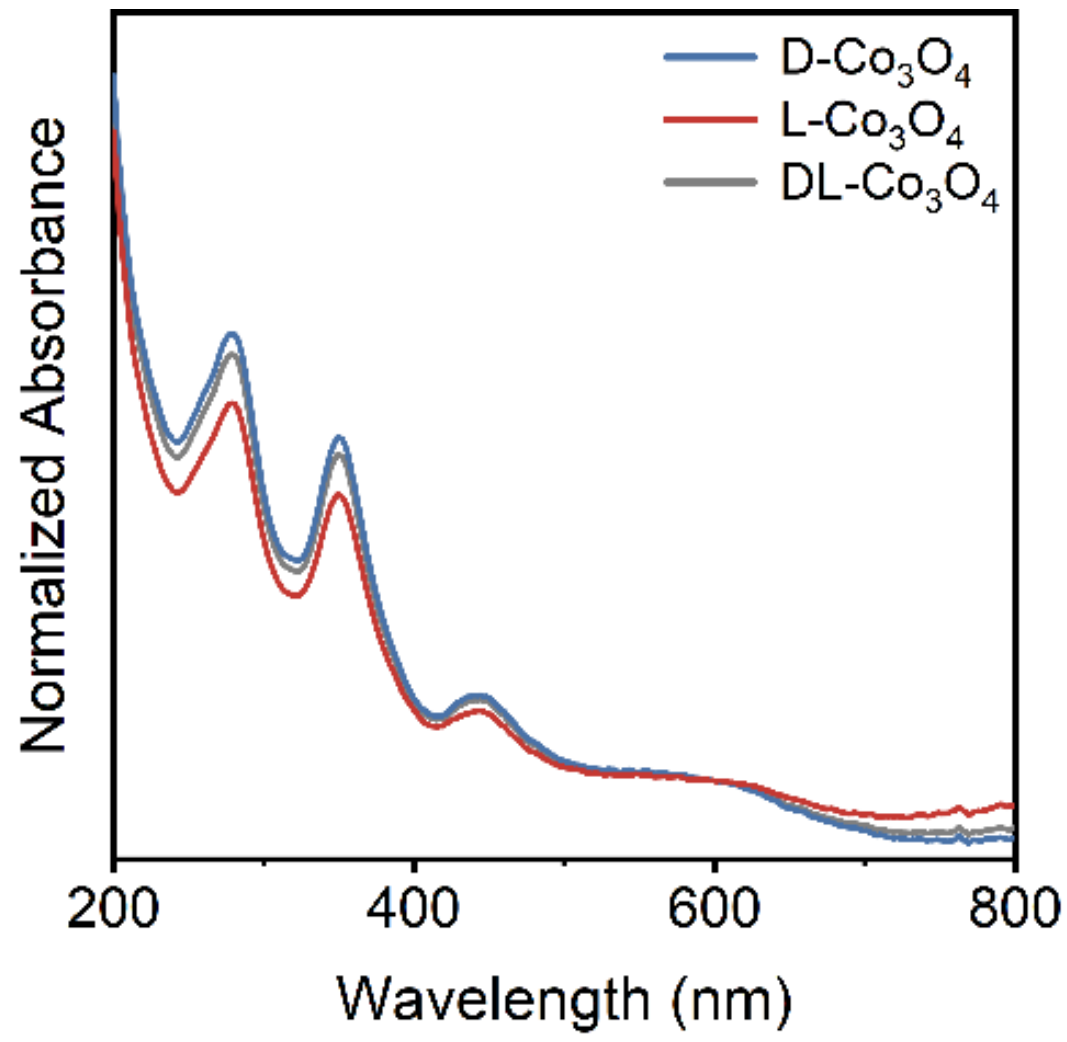

Figure S3. UV-visible spectra of Co_3O_4 nanoparticles stabilized by D-Cys, L-Cys, and DL-Cys.

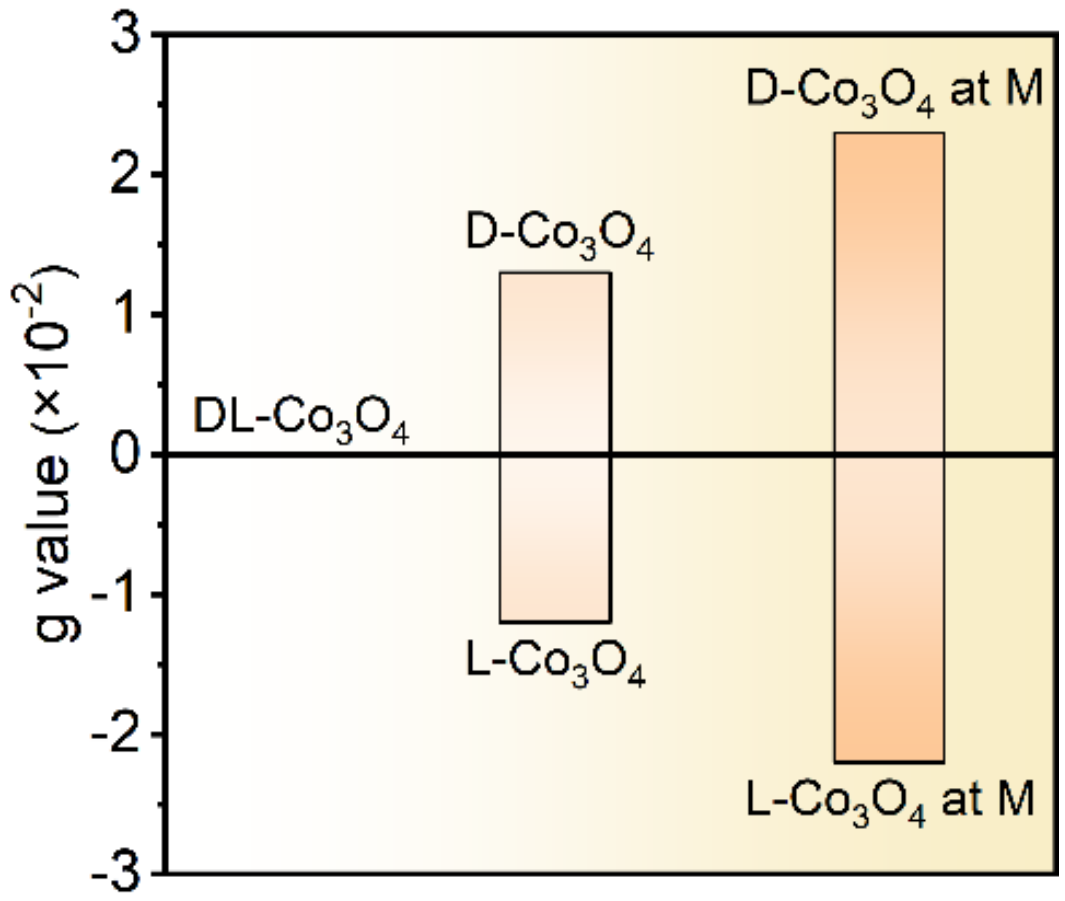

Figure S4. Comparison of the *g* values of chiral Co_3O_4 at the 550 nm CD band.

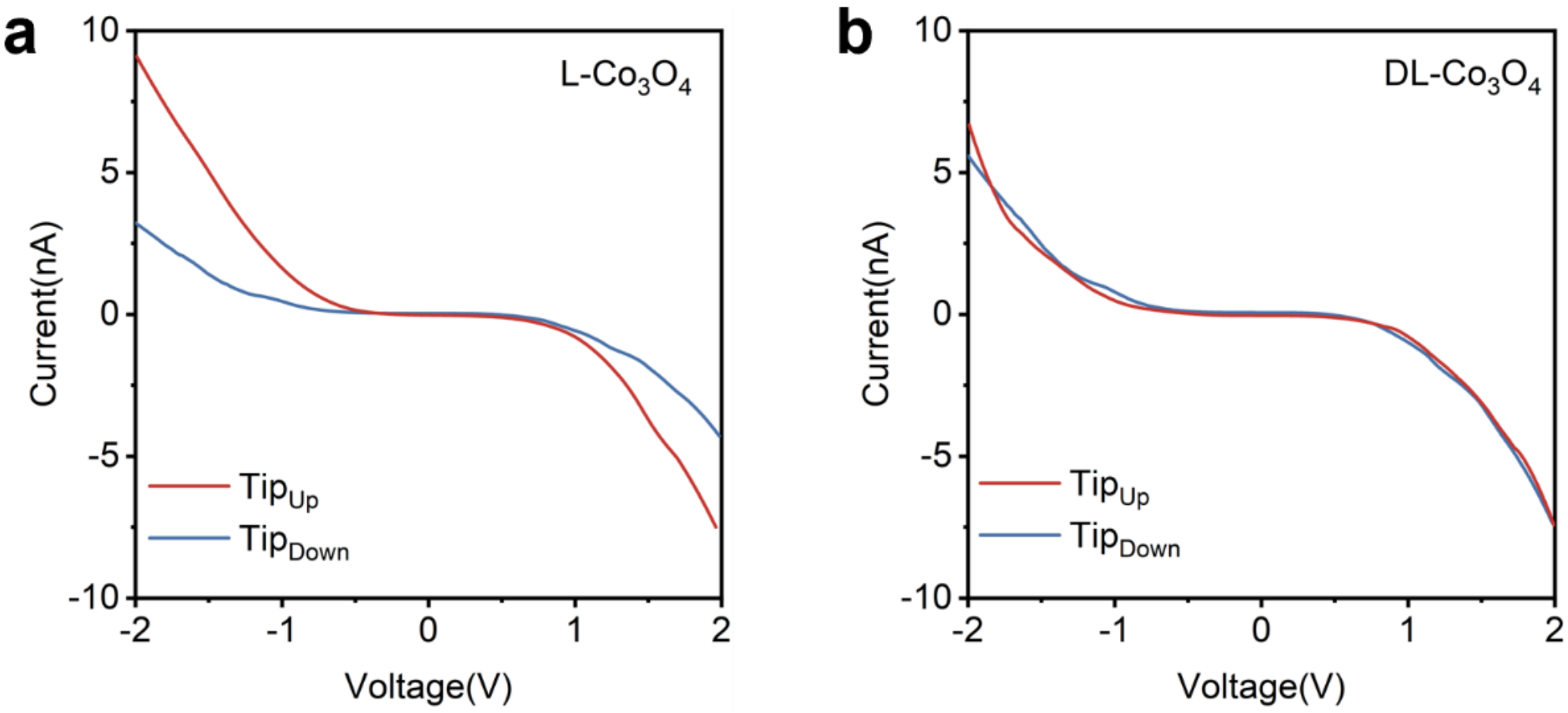

Figure S5. Averaged *I–V* curves obtained for (a) L-Co_3O_4 and (b) DL-Co_3O_4. The tip is magnetized in the up and down.

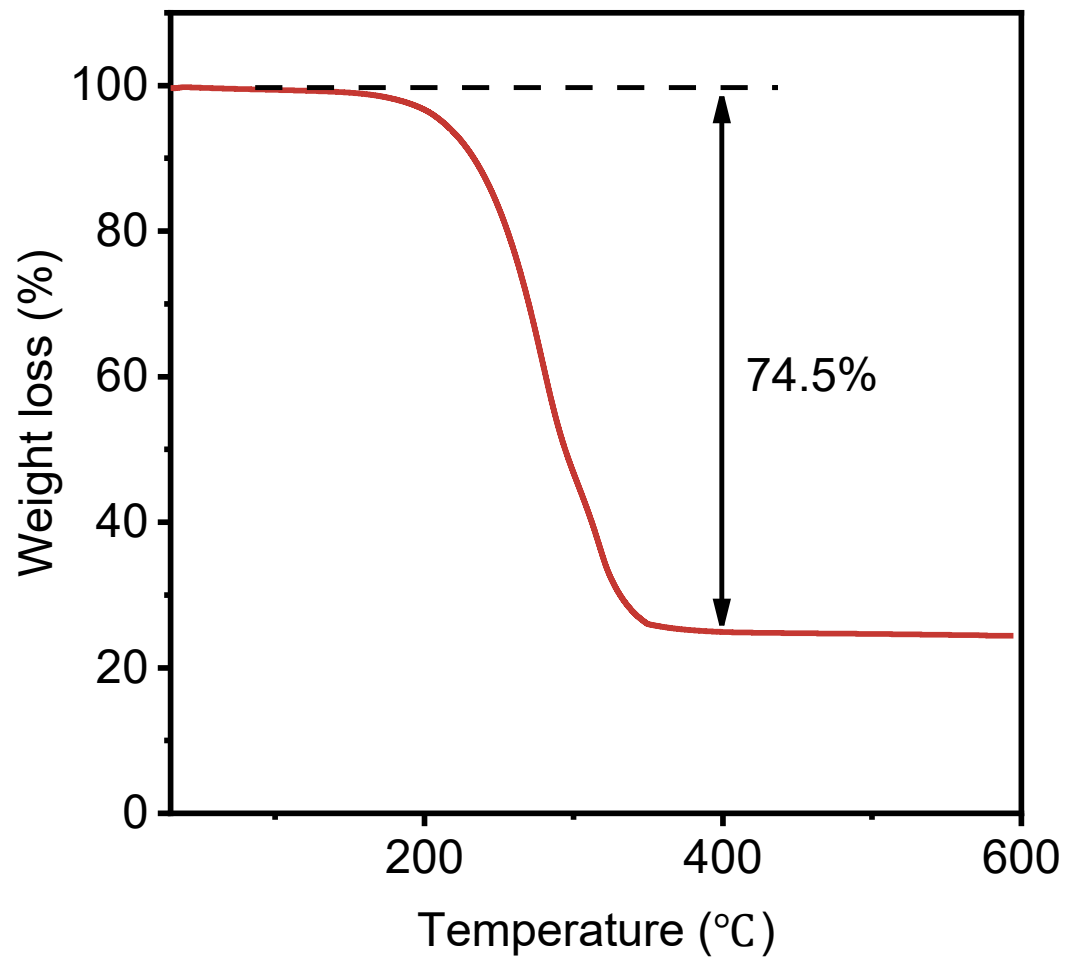

Figure S6. TGA curves of C/S.

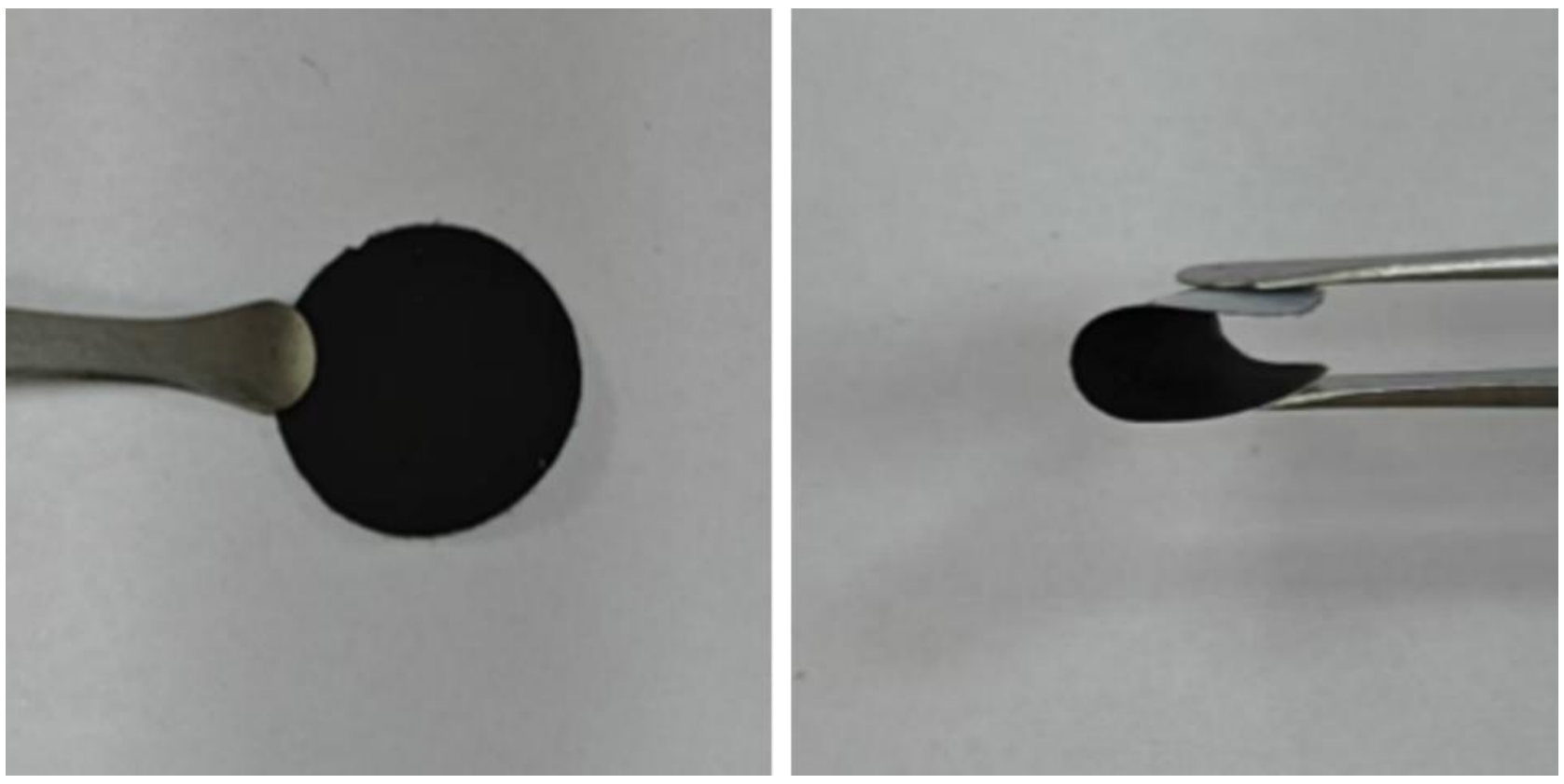

Figure S7. The modified separator.

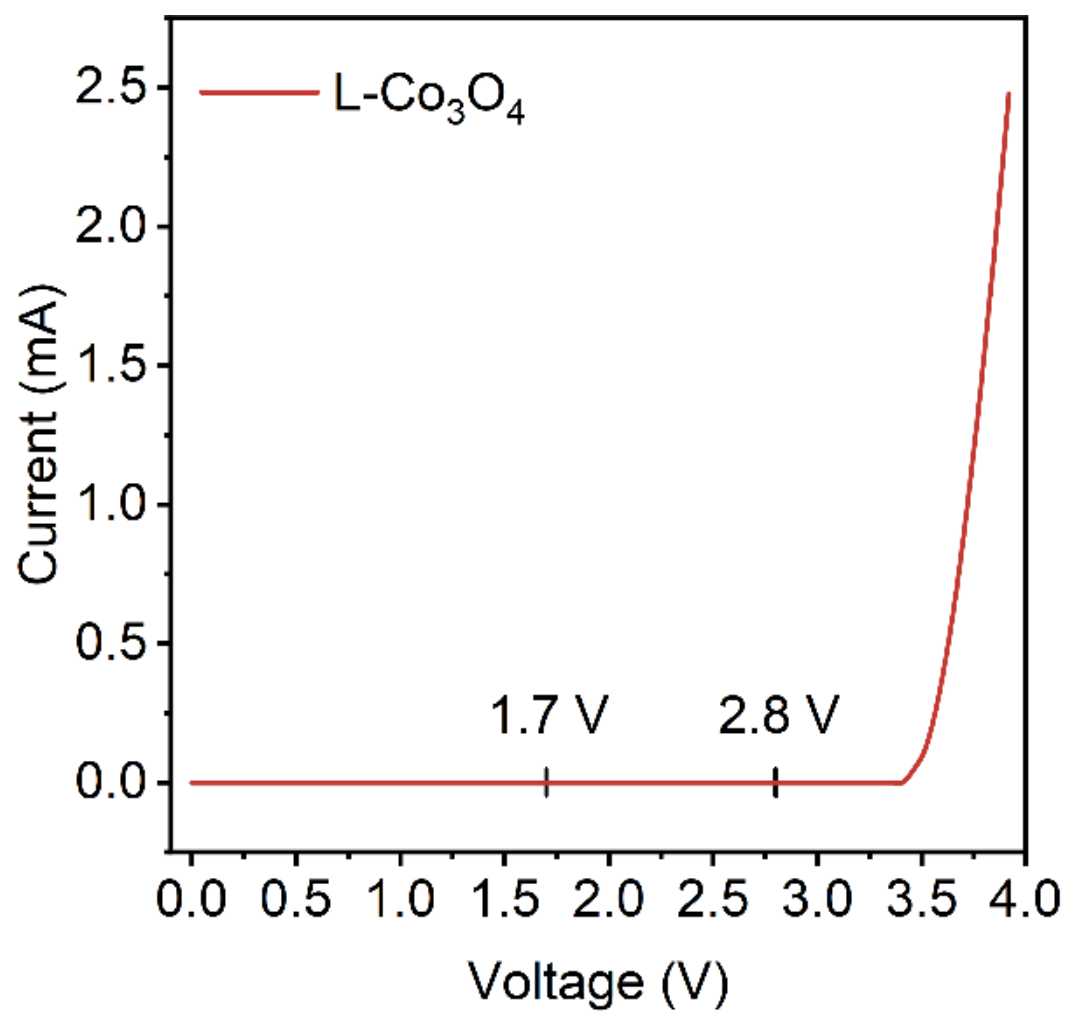

Figure S8. LSV curve of L-Co_3O_4 measured in the Li–S battery electrolyte to evaluate its electrochemical stability within the operating voltage window.

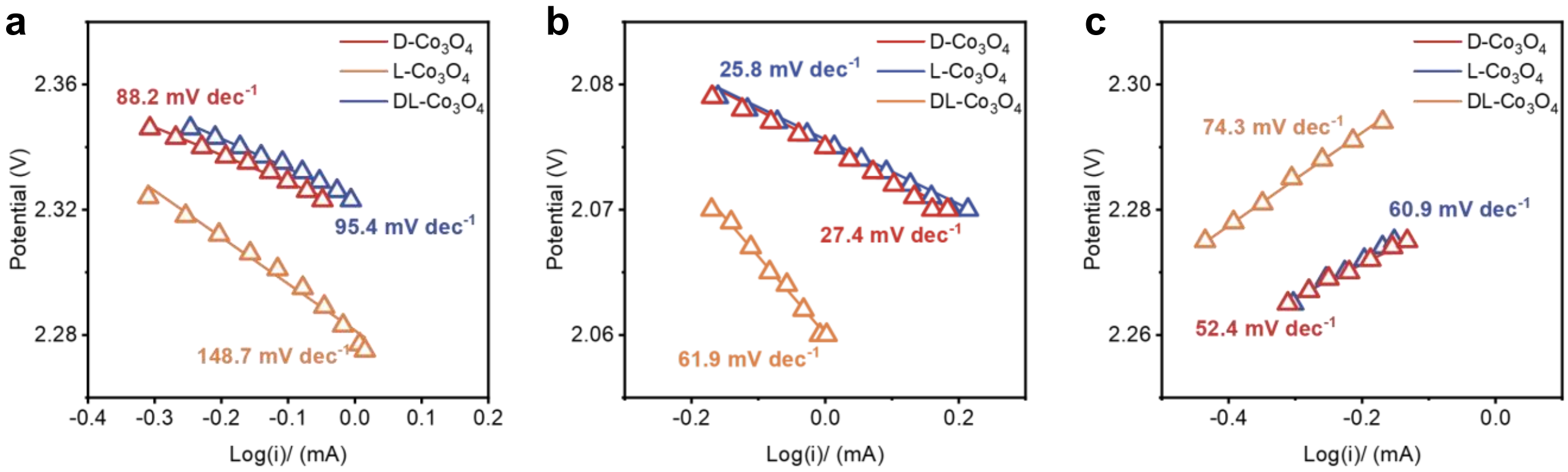

Figure S9. Tafel plots of the three redox peaks (A, B, and C).

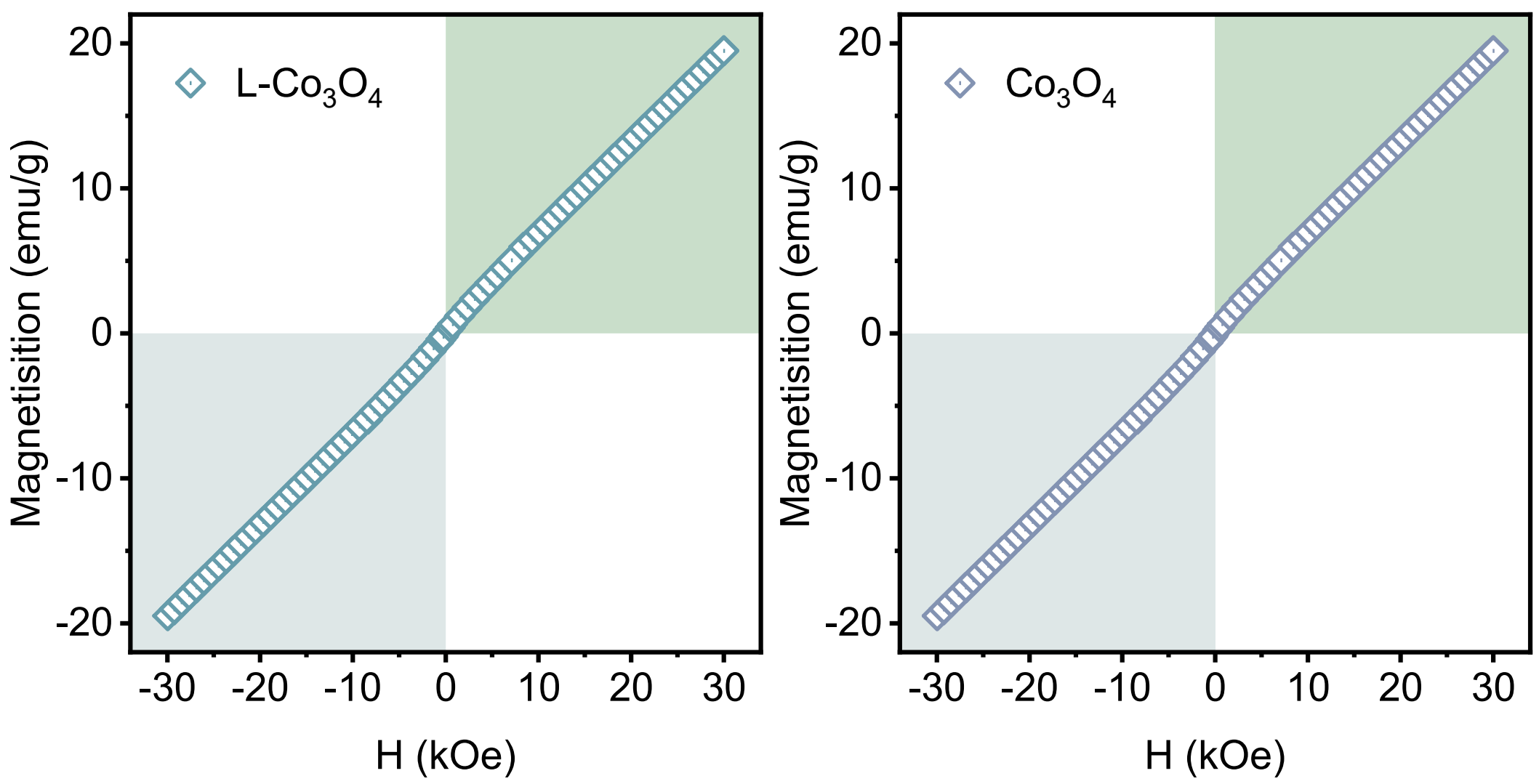

Figure S10. Magnetization hysteresis loop at 300 K indicating the paramagnetic properties of the NPs.

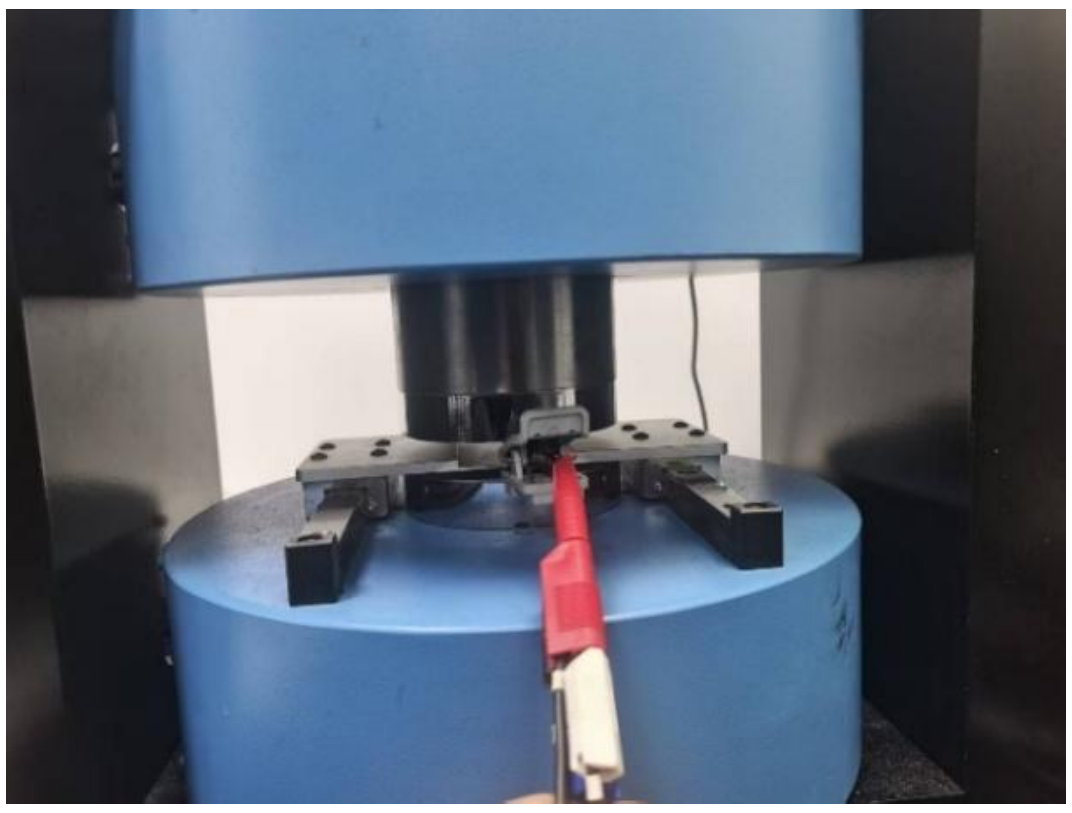

Figure S11. Schematic illustration of the magnetic field generation device.

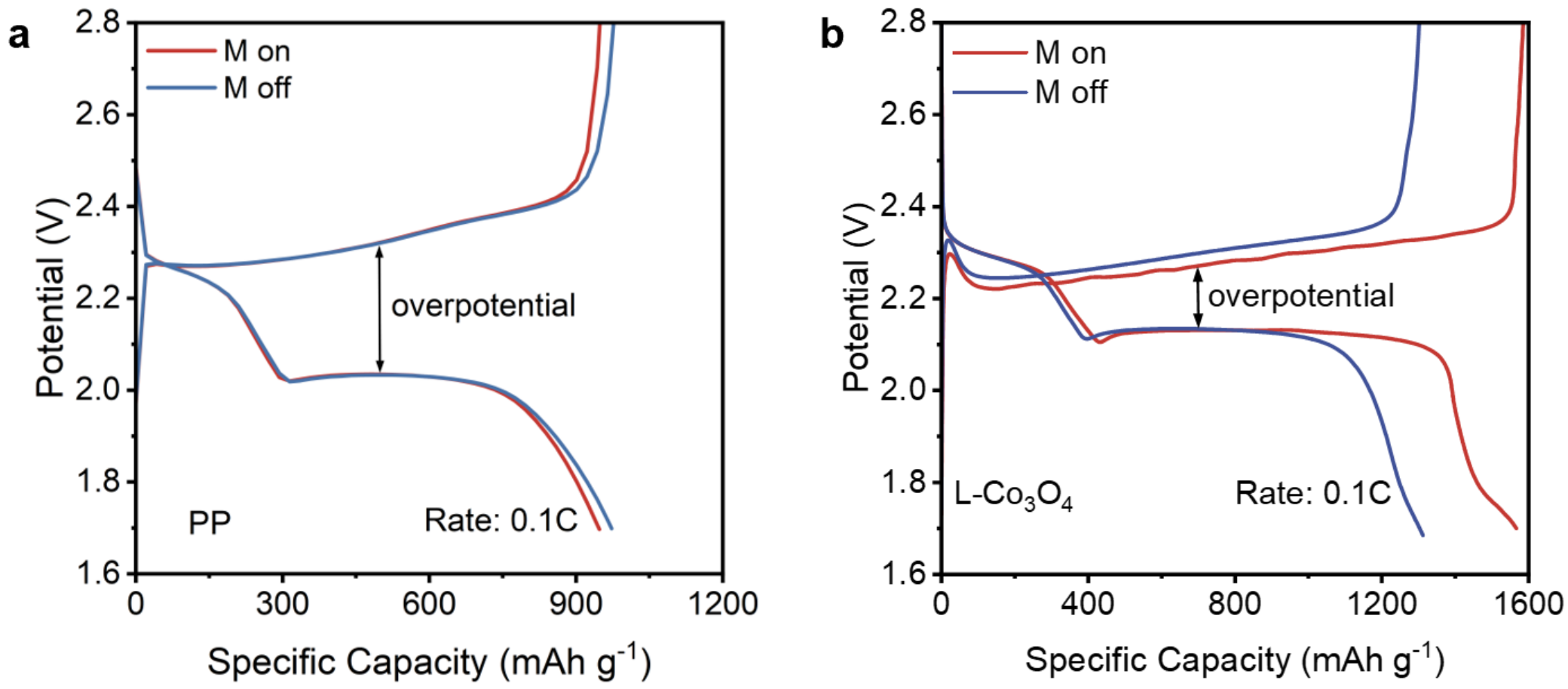

Figure S12. Initial galvanostatic charge–discharge curves of batteries using (a) untreated PP separators and (b) L-Co_3O_4-modified separators under identical conditions before and after applying an external magnetic field.

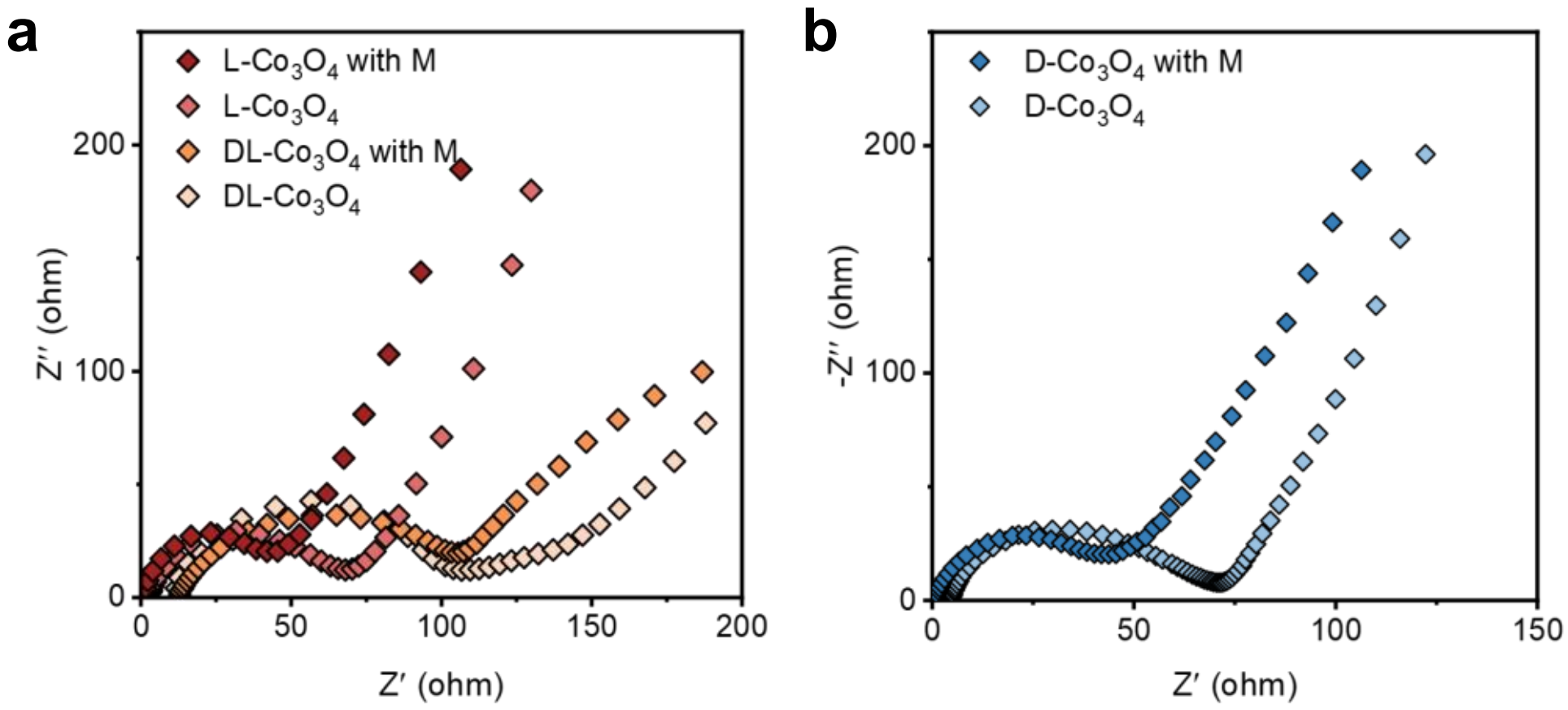

Figure S13. Nyquist plots of D-Co_3O_4 batteries with and without an external magnetic field.

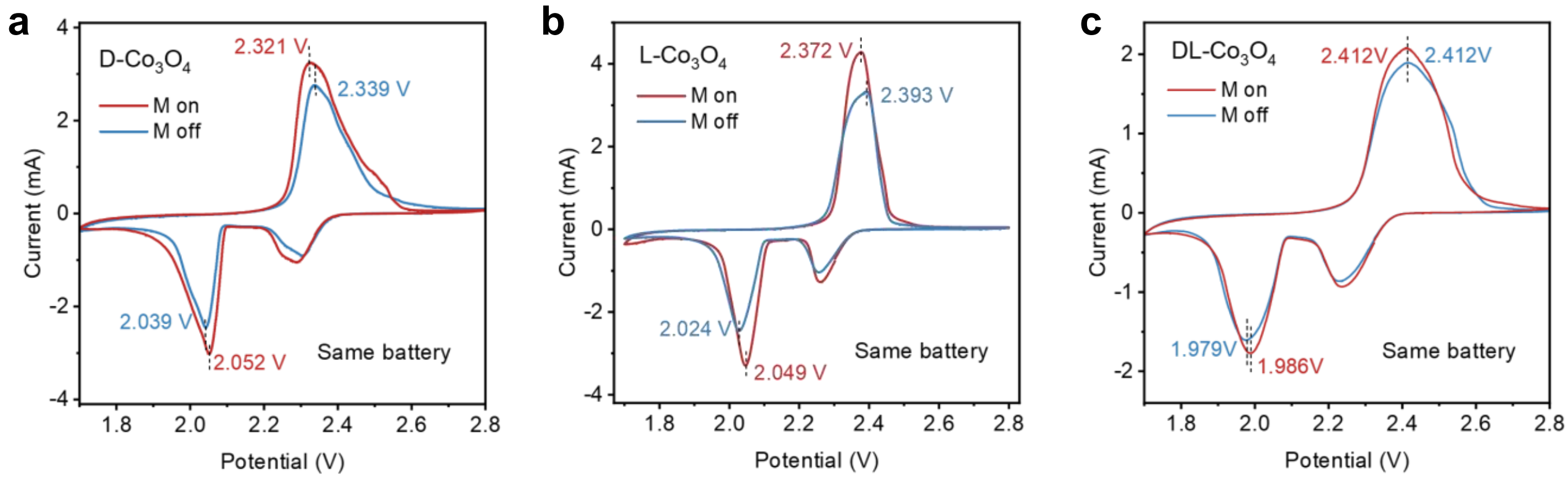

Figure S14. CV curves of D-Co_3O_4, L-Co_3O_4, and DL-Co_3O_4.

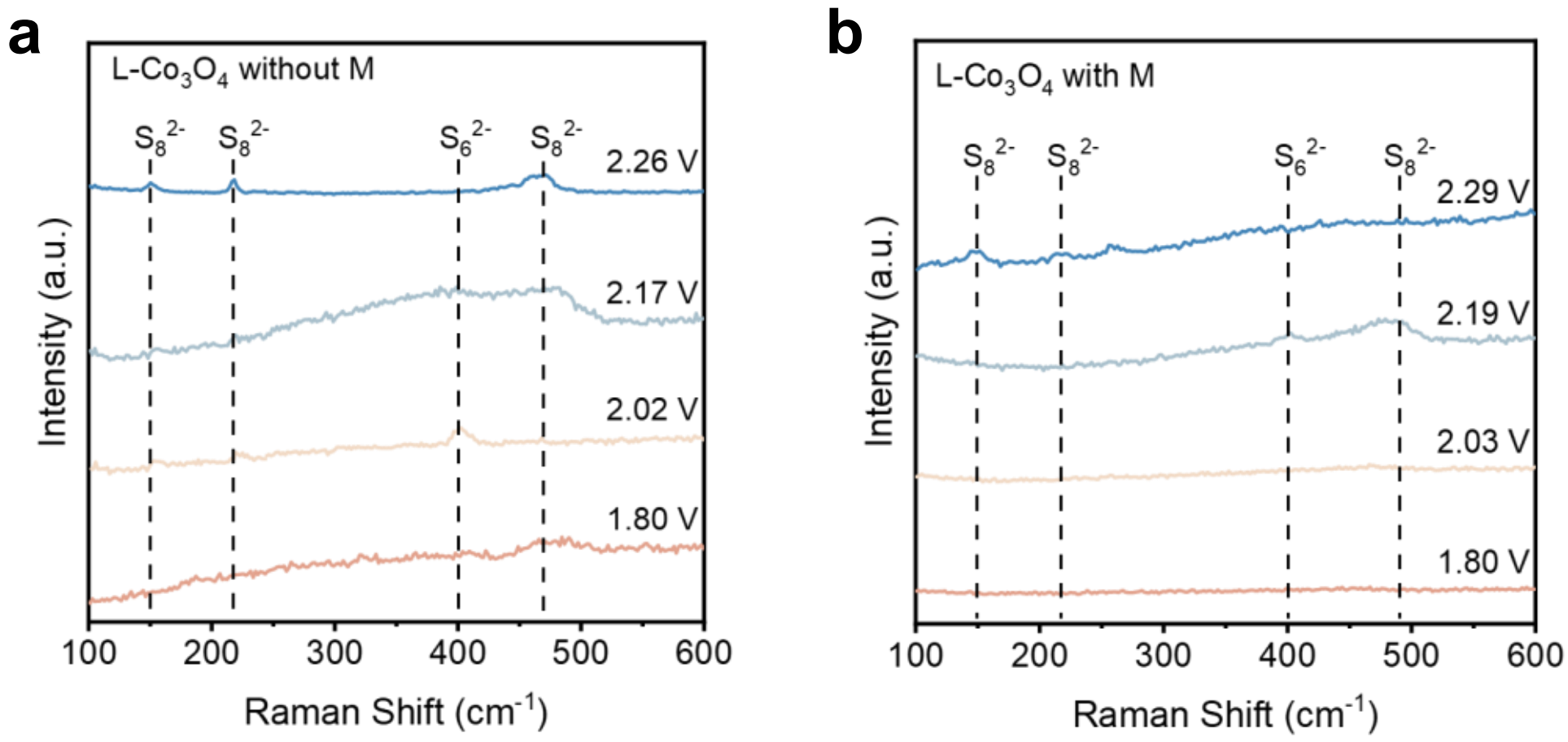

Figure S15. Raman spectra of L-Co_3O_4 without M and L-Co_3O_4 with M batteries.

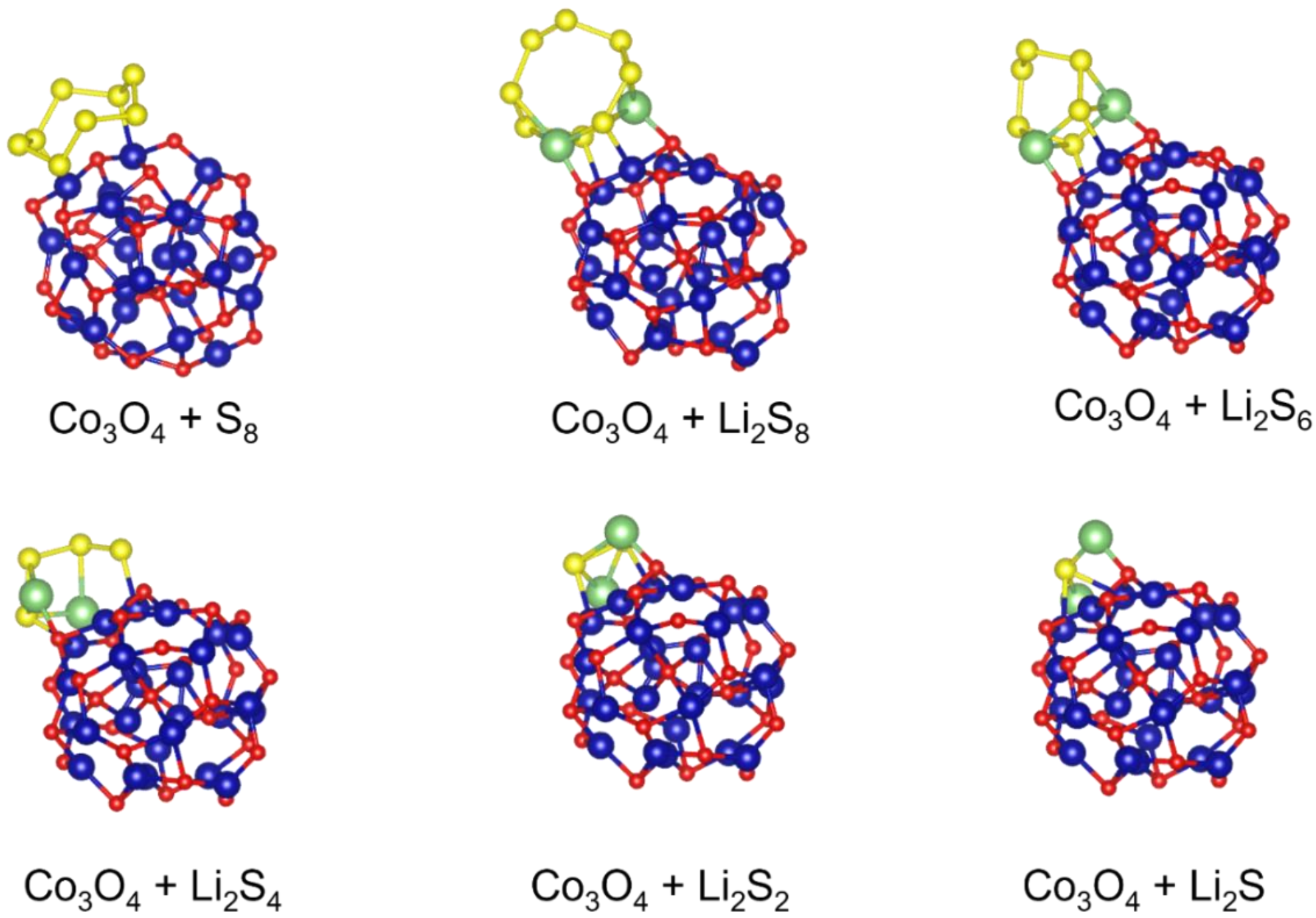

Figure S16. Optimized configurations of adsorbed Li_2S_x on Co_3O_4.

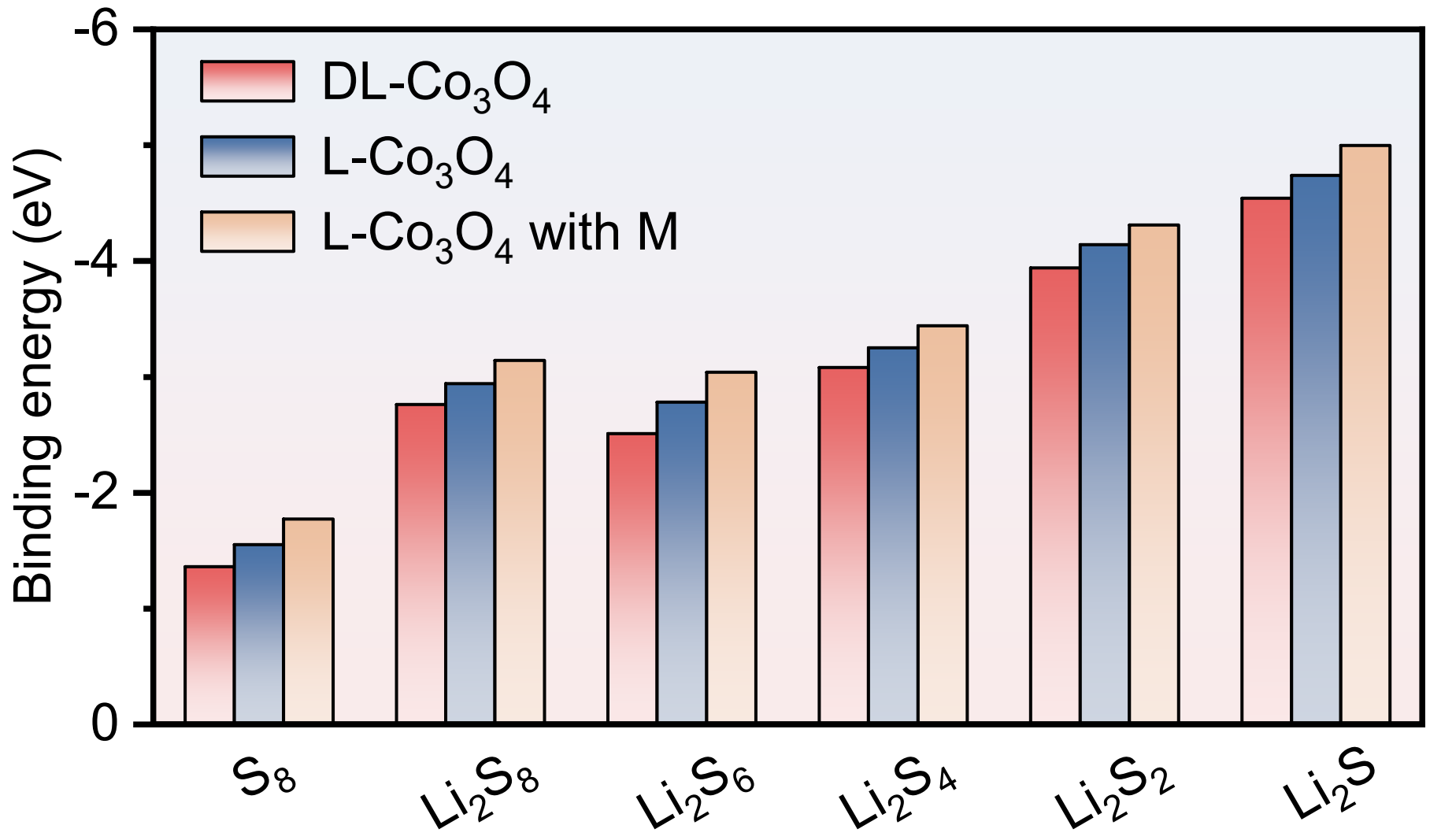

Figure S17. Calculated adsorption energies of DL-Co_3O_4, L-Co_3O_4, and L-Co_3O_4 with M catalysts for S species.
